# Topological Noetherianity of polynomial functors II: base rings with Noetherian spectrum

**DOI:** 10.1007/s00208-022-02386-9

**Published:** 2022-03-19

**Authors:** Arthur Bik, Alessandro Danelon, Jan Draisma

**Affiliations:** 1 University of Bern, Bern, Switzerland; 2 MPI for Mathematics in the Sciences, Leipzig, Germany; 3 Eindhoven University of Technology, Eindhoven, The Netherlands

## Abstract

In a previous paper, the third author proved that finite-degree polynomial functors over infinite fields are topologically Noetherian. In this paper, we prove that the same holds for polynomial functors from free *R*-modules to finitely generated *R*-modules, for any commutative ring *R* whose spectrum is Noetherian. As Erman–Sam–Snowden pointed out, when applying this with $R={{\,\mathrm{{\mathbb Z}}\,}}$ to direct sums of symmetric powers, one of their proofs of a conjecture by Stillman becomes characteristic-independent. Our paper advertises and further develops the beautiful but not so well-known machinery of polynomial laws. In particular, to any finitely generated *R*-module *M* we associate a topological space, which we show is Noetherian when ${{\,\mathrm{Spec}\,}}(R)$ is; this is the degree-zero case of our result on polynomial functors.

## Introduction and main theorem

### Summary

A polynomial functor over an infinite field *K* is a functor *P* from the category of finite-dimensional *K*-vector spaces to itself such that for any two finite-dimensional vector spaces *V*, *W* the map $P_{V,W}:{{\,\mathrm{Hom}\,}}(V,W) \rightarrow {{\,\mathrm{Hom}\,}}(P(V),P(W))$ is a polynomial map. In many respects, polynomial functors behave like univariate polynomials: they can be added (direct sums), multiplied (tensor products), and composed; they are direct sums of unique homogeneous polynomial functors of degrees $0,1,2,\ldots$; and—for the theory that we are about to develop quite importantly—they can be shifted by a constant: if *P* is a polynomial functor and *U* a constant vector space, then the functor ${{\,\mathrm{Sh}\,}}_U(P)$ that assigns to *V* the vector space $P(U \oplus V)$ and to $\varphi \in {{\,\mathrm{Hom}\,}}_K(V,W)$ the linear map $P({{\,\mathrm{id}\,}}_U \oplus \,\varphi )$ is a polynomial functor. Furthermore, if *P* has finite degree, which we will always require, then—much like a univariate polynomial and its shift by a constant—${{\,\mathrm{Sh}\,}}_U(P)$ has the same degree, and the top-degree homogeneous components of *P* and ${{\,\mathrm{Sh}\,}}_U(P)$ are canonically isomorphic.

From a different perspective, polynomial functors are the ambient spaces of “${{\,\mathrm{GL}\,}}_\infty$-equivariant algebraic geometry”, a research area which has seen much activity over the last years. A closed subset of *P* is a rule *X* that assigns to a vector space *V* a Zariski-closed subset *X*(*V*) of *P*(*V*) in such a manner that for each $\varphi \in {{\,\mathrm{Hom}\,}}(U,V)$, the linear map $P_{U,V}(\varphi )$ maps *X*(*U*) into *X*(*V*). In earlier work [8], the third author showed that if *P* has finite degree, then it is Noetherian in the sense that any descending chain of closed subsets $P \supseteq X_1 \supseteq X_2 \supseteq \cdots$ eventually stabilises. This was used in work by Erman–Sam–Snowden [11, 13, 14] and by Draisma–Lasoń–Leykin [9] in new proofs of the conjecture by Stillman that the projective dimension of a homogeneous ideal that is generated by a fixed number of forms of a fixed degree is uniformly bounded independently of the number of variables [20, Problem 3.14]. In this context, Erman–Sam–Snowden asked whether the Noetherianity of polynomial functors also holds over ${{\,\mathrm{{\mathbb Z}}\,}}$; this would show that their proof of Stillman’s conjecture yields bounds that are independent of the characteristic, just like another proof by Erman–Sam–Snowden [11] and the original proof by Ananyan–Hochster [2].

In this paper, we settle Erman–Sam–Snowden’s question in the affirmative. Indeed, rather than working over ${{\,\mathrm{{\mathbb Z}}\,}}$, we will work over a ring *R* whose spectrum is Noetherian—this turns out to be precisely the setting where topological Noetherianity also holds for polynomial functors.

So let *R* be a ring (commutative with 1). In Sect. 3 we will review the notion of *polynomial laws* from an *R*-module *M* to an *R*-module *N*. In the special case where $N=R$, these polynomial laws form a graded ring *R*[*M*] (see Sect. 3.2), where the notation is chosen to resemble that for the coordinate ring of an affine variety. This ring will be used in Sect. 4 to define a topological space ${{\,\mathrm{{\mathbb A}}\,}}_M$, in such a manner that any polynomial law $\varphi :M \rightarrow N$ yields a continuous map, also denoted $\varphi$, from ${{\,\mathrm{{\mathbb A}}\,}}_M \rightarrow {{\,\mathrm{{\mathbb A}}\,}}_N$. To be precise, ${{\,\mathrm{{\mathbb A}}\,}}_M$ is a topological space over the category ${{\,\mathrm{\mathbf {Dom}_R}\,}}$ of *R*-domains with *R*-algebra monomorphisms. Here a topological space over a category $\mathbf {C}$ is not a single set, but a functor from $\mathbf {C}$ equipped with the notions of elements and (closed) subsets, and we let all definitions related to usual topological spaces stated in terms of their elements and (closed) subsets carry over to this setting; see Definition 28 for details.

If *M* is freely generated by *n* elements, then *R*[*M*] is the polynomial ring $R[x_1,\ldots ,x_n]$ and the poset of closed sets in ${{\,\mathrm{{\mathbb A}}\,}}_M$ is the same as that in the spectrum of *R*[*M*]. In general, however, we do not completely understand the relation between ${{\,\mathrm{{\mathbb A}}\,}}_M$ and the spectrum of *R*[*M*] (see Remark 41), and we work with the former rather than the latter. The following result is a topological version of Hilbert’s basis theorem in this setting.

#### Proposition 1

If *R* has a Noetherian spectrum and *M* is a finitely generated *R*-module, then the topological space ${{\,\mathrm{{\mathbb A}}\,}}_M$ over ${{\,\mathrm{\mathbf {Dom}_R}\,}}$ is Noetherian.

Interestingly, it is not true that if *R* is Noetherian and *M* is finitely generated, then *R*[*M*] is Noetherian (see Example 23), so “topologically Noetherian” is the most natural setting here. A special case of the theorem (taking *M* free of rank 1) is that if *R* has a Noetherian spectrum, then so does the polynomial ring *R*[*x*]. This special case, a topological version of Hilbert’s basis theorem, is easy and well-known; e.g., it also follows from [12, Theorem 1.1] with a trivial group *G*.

Following [22], in Sect. 5 we will recall the notion of polynomial functors from the category ${{\,\mathrm{\mathbf {fgfMod}_R}\,}}$ of finitely generated free *R*-modules to the category ${{\,\mathrm{\mathbf {Mod}_R}\,}}$ of *R*-modules. These polynomial functors form an Abelian category. The subcategory of polynomial functors from ${{\,\mathrm{\mathbf {fgfMod}_R}\,}}$ to the category ${{\,\mathrm{\mathbf {fgMod}_R}\,}}$ of finitely generated, but not necessarily free, *R*-modules is not an Abelian subcategory when *R* is not Noetherian, but it is closed under taking quotients, and this will suffice for our purposes.

Given a polynomial functor $P:{{\,\mathrm{\mathbf {fgfMod}_R}\,}}\rightarrow {{\,\mathrm{\mathbf {fgMod}_R}\,}}$, a closed subset of ${{\,\mathrm{{\mathbb A}}\,}}_P$ is a rule *X* that assigns to each finitely generated free *R*-module *U* a closed subset *X*(*U*) of ${{\,\mathrm{{\mathbb A}}\,}}_{P(U)}$ such that the continuous map corresponding to the polynomial law

$$\begin{aligned} {{\,\mathrm{Hom}\,}}(U,V) \times P(U) \rightarrow P(V),\quad (\varphi ,p) \mapsto P_{U,V}(\varphi )(p) \end{aligned}$$

maps the pre-image of *X*(*U*) under the projection on *P*(*U*) in ${{\,\mathrm{{\mathbb A}}\,}}_{{{\,\mathrm{Hom}\,}}(U,V) \times P(U)}$ into *X*(*V*) (see Sect. 5.8 for details). If *Y* is a second such rule, then we say that *X* is a subset of *Y* if *X*(*U*) is a subset of *Y*(*U*) for each $U \in {{\,\mathrm{\mathbf {fgfMod}_R}\,}}$. Our main result, then, is the following.

#### Theorem 2

Let *R* be a commutative ring whose spectrum is a Noetherian topological space and let *P* be a finite-degree polynomial functor ${{\,\mathrm{\mathbf {fgfMod}_R}\,}}\rightarrow {{\,\mathrm{\mathbf {fgMod}_R}\,}}$. Then every descending chain $X_1 \supseteq X_2 \supseteq \ldots$ of closed subsets of ${{\,\mathrm{{\mathbb A}}\,}}_P$ stabilises: for all sufficiently large *n* we have $X_n=X_{n+1}$.

Proposition 1 is the special case of Theorem 2 where the polynomial functor has degree 0, i.e., sends each *U* to a fixed module *M* and each morphism to the identity ${{\,\mathrm{id}\,}}_M$. Proposition 1 will be proved first, as a base case in an inductive proof of Theorem 2.

### Structure of the paper

In Sect. 2, we establish and recall certain basic results. In Sect. 3 we define polynomial laws and the coordinate ring of a module over a ring. Section 4 is devoted to the topological space ${{\,\mathrm{{\mathbb A}}\,}}_M$. Here we prove Proposition 1, the first fundamental fact needed for our inductive proof of Theorem 2.

Then, in Sect. 5 we recall the definition a polynomial functor *P* over a ring and several of its properties. Among these is the Friedlander–Suslin lemma that yields equivalences of Abelian categories between polynomial functors ${{\,\mathrm{\mathbf {fgfMod}_R}\,}}\rightarrow {{\,\mathrm{\mathbf {fgMod}_R}\,}}$ of degree $\le d$ and finitely generated modules for the non-commutative *R*-algebra $R[{{\,\mathrm{End}\,}}(U)]_{\le d}^*$ (called the Schur algebra) for any $U \in {{\,\mathrm{\mathbf {fgfMod}_R}\,}}$ of rank $\ge d$. We also prove the second fundamental fact needed for Theorem 2: if *R* is a domain and *P* a polynomial functor from ${{\,\mathrm{\mathbf {fgfMod}_R}\,}}$ to ${{\,\mathrm{\mathbf {Mod}_R}\,}}$ such that ${{\,\mathrm{Frac}\,}}(R) \otimes P$ is irreducible, then ${{\,\mathrm{Frac}\,}}(R/\mathfrak {p}) \otimes P$ is irreducible for all primes $\mathfrak {p}$ in some open dense subset of ${{\,\mathrm{Spec}\,}}(R)$. This is an incarnation of the philosophy in representation theory that irreducibility is a generic condition.

Finally, in Sect. 6 we prove Theorem 2. The global proof strategy is as follows: we show that the induction steps in [8], where Theorem 2 is proved when *R* is an infinite field, can be made global in the sense that they hold for ${{\,\mathrm{Frac}\,}}(R/\mathfrak {p})$ for all $\mathfrak {p}$ in some open dense subset of ${{\,\mathrm{Spec}\,}}(R)$; and then we use Noetherian induction on ${{\,\mathrm{Spec}\,}}(R)$ to deal with the remaining primes $\mathfrak {p}$. The details of this approach are a quite subtle and beautiful.

The big picture is depicted in the following diagram:
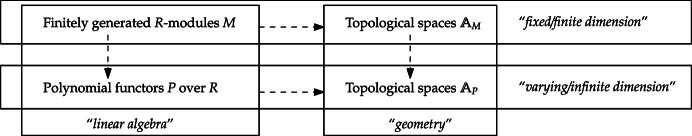


Building on the notion of finitely generated *R*-modules, on the left we pass to polynomial functors over *R*. Here many results carry over, such as the fact that the rank is a semicontinuous function on ${{\,\mathrm{Spec}\,}}(R)$; see Proposition 54. We regard this as “linear algebra in varying dimensions”. In the other direction, we construct the topological space ${{\,\mathrm{{\mathbb A}}\,}}_M$ and enter the realm of algebraic geometry; the closed subsets generalise affine algebraic varieties. Finally, both constructs come together in the construction of the topological space associated to a polynomial functor *P*. Here we use both results from the “linear algebra” of polynomial functors, such as Friedlander–Suslin’s lemma, and results about the topological spaces ${{\,\mathrm{{\mathbb A}}\,}}_M$, to prove that ${{\,\mathrm{{\mathbb A}}\,}}_P$ is Noetherian. Furthermore, we establish the fundamental result that the dimension function of a closed subset of ${{\,\mathrm{{\mathbb A}}\,}}_P$ depends on primes in ${{\,\mathrm{Spec}\,}}(R)$ in a constructible manner; see Proposition 86.

### A class of applications

Our original motivation for this paper is the following: let *P*, *Q* be (finite-degree) polynomial functors from the category of finitely generated free ${{\,\mathrm{{\mathbb Z}}\,}}$-modules to itself and let $\alpha :Q \rightarrow P$ be a polynomial transformation; see Definition 46. Define the closed subset *X* of ${{\,\mathrm{{\mathbb A}}\,}}_P$ as the closure of the image of $\alpha$. Specifically, for a natural number *n*, the pull-back along $\alpha _{{{\,\mathrm{{\mathbb Z}}\,}}^n}$ defines a ring homomorphism ${{\,\mathrm{{\mathbb Z}}\,}}[P({{\,\mathrm{{\mathbb Z}}\,}}^n)] \rightarrow {{\,\mathrm{{\mathbb Z}}\,}}[Q({{\,\mathrm{{\mathbb Z}}\,}}^n)]$, and $X({{\,\mathrm{{\mathbb Z}}\,}}^n)$ is the closed subset of ${{\,\mathrm{Spec}\,}}{{\,\mathrm{{\mathbb Z}}\,}}[P({{\,\mathrm{{\mathbb Z}}\,}}^n)]$ defined by the kernel of that ring homomorphism. Theorem 2 implies the following.

#### Corollary 3

There exists a uniform bound *d* such that for all $n \in {{\,\mathrm{{\mathbb Z}}\,}}_{\ge 0}$ and all fields *K*, $X(K^n) \subseteq K \otimes P({{\,\mathrm{{\mathbb Z}}\,}}^n)$ is defined by polynomials of degree $\le d$.

This corollary has many applications; here is one. If *V* is a finite-dimensional vector space over a field *K* and $T \in V \otimes V \otimes V$ is a tensor, then *T* is said to have *slice rank* $\le r$ if *T* can be written as the sum of *r* terms of the form $\sigma (v \otimes A)$, where $v \in V$ and $A \in V \otimes V$, and $\sigma$ is a cyclic permutation of 1, 2, 3 permuting the tensor factors. If *K* is algebraically closed, then being of slice rank $\le r$ is a Zariski-closed condition [26].

#### Corollary 4

Fix a natural number *r*. There exists a uniform bound *d* such that for all algebraically closed fields *K* and for all $n \in {{\,\mathrm{{\mathbb Z}}\,}}_{\ge 0}$, the variety of slice-rank-$\le r$ tensors in $K^n \otimes K^n \otimes K^n$ is defined by polynomials of degree $\le d$.

The same holds when the number of tensor factors is increased to any fixed number, possibly at the expense of increasing *d*, and similar results hold for the set of cubic forms of bounded q-rank [7] or for the closure of the set of degree-*e* forms of bounded strength in the sense of [2]. We stress, however, that “defined by” is intended in a purely set-theoretic sense. We do not know whether the vanishing ideals of these varieties are generated in bounded degree, even if the field *K* were fixed beforehand.

#### Proof of Corollary 4

Consider the polynomial functor *P* that sends a free ${{\,\mathrm{{\mathbb Z}}\,}}$-module ${{\,\mathrm{{\mathbb Z}}\,}}^n$ to ${{\,\mathrm{{\mathbb Z}}\,}}^n \otimes {{\,\mathrm{{\mathbb Z}}\,}}^n \otimes {{\,\mathrm{{\mathbb Z}}\,}}^n$, and the polynomial functor *Q* that sends ${{\,\mathrm{{\mathbb Z}}\,}}^n$ to ${{\,\mathrm{{\mathbb Z}}\,}}^n \oplus ({{\,\mathrm{{\mathbb Z}}\,}}^n \otimes {{\,\mathrm{{\mathbb Z}}\,}}^n)$. For any *r*-tuple $(\sigma _1,\ldots ,\sigma _r)$ of cyclic permutations of 1, 2, 3 we have a polynomial transformation

$$\begin{aligned} Q^r \rightarrow P, ((v_1,A_1),\ldots ,(v_r,A_r)) \mapsto \sum _{i=1}^r \sigma _i(v_i \otimes A_i), \end{aligned}$$

whose image closure is defined in uniformly bounded degree *e* by Corollary 3. The variety of slice-rank-$\le r$ tensors is the union of these image closures over all *r*-tuples of cyclic permutations, hence defined in degree at most $e \cdot 3^r$, independently of the algebraically closed field and independently of *n*. $\square$

#### Remark 5

Over a field *K* of characteristic zero, the irreducible polynomial functors *P* are precisely the Schur functors, and any polynomial functor is isomorphic to a direct sum of Schur functors. These always admit a ${{\,\mathrm{{\mathbb Z}}\,}}$-form, i.e., a polynomial functor $P_{{{\,\mathrm{{\mathbb Z}}\,}}}$ over ${{\,\mathrm{{\mathbb Z}}\,}}$ such that $K \otimes P_{{{\,\mathrm{{\mathbb Z}}\,}}} \cong P$, which moreover has the property that it maps free ${{\,\mathrm{{\mathbb Z}}\,}}$-modules to free ${{\,\mathrm{{\mathbb Z}}\,}}$-modules [1]. The ${{\,\mathrm{{\mathbb Z}}\,}}$-form need not be unique; e.g., the Schur functor over *K* that maps *V* to its *d*-th symmetric power $S^d V$, comes both from the functor from free ${{\,\mathrm{{\mathbb Z}}\,}}$-modules to free ${{\,\mathrm{{\mathbb Z}}\,}}$-modules that sends *U* to $S^d U$ and from the functor that sends *U* to the sub-${{\,\mathrm{{\mathbb Z}}\,}}$-module of $U^{\otimes d}$ consisting of symmetric tensors. These two functors are non-isomorphic ${{\,\mathrm{{\mathbb Z}}\,}}$-forms. In applications such as the above, where one looks for field-independent bounds, it is important to choose the ${{\,\mathrm{{\mathbb Z}}\,}}$-form that captures the problem of interest.

#### Example 6

Again over $R={{\,\mathrm{{\mathbb Z}}\,}}$, consider the polynomial transformation $\alpha :(S^2)^4 \rightarrow S^4$ that maps a quadruple $(q_1,\ldots ,q_4)$ of quadratic forms to $q_1^2 + \cdots + q_4^2$. Let *X* be the image closure as above. If *K* is algebraically closed of characteristic zero, then $X_K(K^4)$ is a hypersurface in $S^4(K)$ of degree 38475 [3], so the degree bound from Corollary 3 must be at least that large. On the other hand, if *K* is algebraically closed of characteristic 2, then the image of $\alpha$ is just the linear space spanned by all degree-four monomials that are squares, and hence only linear equations are needed to cut out this image.

#### Remark 7

Over algebraically closed fields of positive characteristic, irreducible polynomial functors are still parameterised by partitions, but polynomial functors are no longer semisimple, and the ${{\,\mathrm{{\mathbb Z}}\,}}$-forms from Remark 5 do not always remain irreducible; standard references are [6, 16]. The typical example is that, in characteristic *p*, the functor $S^p$ contains a subfunctor that maps *V* to the linear space of *p*-th powers of elements of *V*.

### Further relations to the literature

The polynomial functors that we study are often referred to as strict polynomial functors in the literature; we will drop the adjective “strict”. We do not know whether the polynomial functors over finite fields studied in [21] admit a similar theory.

Much literature on polynomial functors is primarily concerned with representation theory, whereas our emphasis is on the geometry/commutative algebra of closed subsets in such polynomial functors.

We will use work of Roby on polynomial laws [22] and work of Touzé on polynomial functors [27]—but indeed only more elementary parts of their work, such as the generalisation of Friedlander–Suslin’s [15, Theorem 3.2] to general base rings *R*; see [27, Théorème 7.2].

The paper [14] establishes finiteness results for (cone-stable and weakly upper semi-continuous) ideal invariants in polynomial rings over a fixed field. As Erman pointed out to us, at least part of their results carry over to arbitrary base rings with Noetherian spectrum. In particular, Erman–Sam–Snowden establish the Noetherianity of a space $Y_{{{\,\mathrm{\mathbf {d}}\,}}}$ that parameterises homogeneous ideals generated in degrees ${{\,\mathrm{\mathbf {d}}\,}}=(d_1,\ldots ,d_r)$. While they work with certain limit spaces, the “functor analogue” of their $Y_{{{\,\mathrm{\mathbf {d}}\,}}}$ in our setting would be a functor from ${{\,\mathrm{\mathbf {fgfMod}_R}\,}}$ to the category of functors from ${{\,\mathrm{\mathbf {Dom}_R}\,}}$ to sets that sends a finitely generated free *R*-module $U=R^n$ to the functor that maps an *R*-domain *D* to the set of ${{\,\mathrm{GL}\,}}_n(D)$-orbits of ideals in $R[x_1,\ldots ,x_n]$ generated by homogeneous polynomials of degrees $d_1,\ldots ,d_r$. Then $Y_{{{\,\mathrm{\mathbf {d}}\,}}}$ admits a surjective map from the space ${{\,\mathrm{{\mathbb A}}\,}}_{S^{d_1} \oplus \cdots \oplus S^{d_r}}$—a functor from ${{\,\mathrm{\mathbf {fgfMod}_R}\,}}$ to functors from ${{\,\mathrm{\mathbf {Dom}_R}\,}}$ to topological spaces, and one can give $Y_{{{\,\mathrm{\mathbf {d}}\,}}}$ the quotient topology. Theorem 2 implies that $Y_{{{\,\mathrm{\mathbf {d}}\,}}}$ is then Noetherian, provided that ${{\,\mathrm{Spec}\,}}(R)$ is Noetherian.

Our work does not say much about Noetherianity of the coordinate rings $R[{{\,\mathrm{{\mathbb A}}\,}}_P]$, let alone about Noetherianity of finitely generated modules over them. Currently, these much stronger results are known only when *R* is a field of characteristic zero and *P* is either a direct sum of copies of $S^1$ [23, 24] or $P=S^2$ or $P=\bigwedge ^2$ [19] or $P=S^1 \oplus S^2$ or $P=S^1 \oplus \bigwedge ^2$ [25].

Like Ananyan–Hochster’s work [2], recent work by Kazhdan and Ziegler [17, 18] implies that polynomials of high strength, and high-strength sequences of polynomials, behave very much like generic polynomials or sequences. Like Corollary 3, their results are uniform in the characteristic of the field. But the route that Kazhdan and Ziegler take is entirely different: first a theorem is proved over finite fields by algebraic-combinatorial means, with uniform constants that do not depend on the finite field, and then model theory is used to transfer the result to arbitrary algebraically closed fields.

In [4] it is shown that in any closed subset of the polynomial functor $S^d$ defined over ${{\,\mathrm{{\mathbb Z}}\,}}$, the strength of polynomials over a ground field of characteristic 0 or characteristic $>d$ is uniformly bounded from above. While of a similar flavour as Corollary 3, that result—in which the restriction on the characteristic cannot be removed—does not follow from our current work. Far-reaching generalisations of [4], but only over fields of characteristic zero, are the topic of the forthcoming preprint [5].

## Preliminaries

### Rings and algebras

Throughout the paper, all rings are commutative and with 1 and ring homomorphisms are required to be unital. We fix a ring *R*, and if $\mathfrak {p}$ is a prime ideal in *R*, then we write $K_\mathfrak {p}$ for the fraction field of the domain $R/\mathfrak {p}$. If *R* is a domain, then we write $K:=K_{(0)}$ for the fraction field of *R*.

An *R*-algebra is an (unless otherwise stated) commutative ring with a homomorphism from *R* into it; an *R*-algebra homomorphisms from an *A* to *B* is a ring homomorphism $A \rightarrow B$ such that composition of the homomorphisms $R \rightarrow A \rightarrow B$ is the prescribed homomorphism $R \rightarrow B$. Except where specified otherwise, tensor products are over *R*, ${{\,\mathrm{Hom}\,}}(U,V)$ is the *R*-module of *R*-module homomorphisms from *U* to *V*, and $U^*={{\,\mathrm{Hom}\,}}(U,R)$. We use the terms *R*-domain and *R*-field for *R*-algebras that, as rings, are domains and fields, respectively.

### From finitely generated to free modules

The following lemma, which we will later generalise to polynomial functors, is well-known; we give a proof for completeness.

#### Lemma 8

Let *R* be a domain, let *M* be a finitely generated *R*-module, and let *N* be a submodule of *M*. Then there exists a nonzero $r \in R$ and elements $v_1,\ldots ,v_n \in N$ such that $R[1/r] \otimes N$ is a finitely generated free submodule of $R[1/r] \otimes M$ with basis $1 \otimes v_1, \ldots , 1 \otimes v_n$, and such that $R[1/r] \otimes M$ is the direct sum of $R[1/r] \otimes N$ and another free *R*[1/*r*]-module.

Note that tensoring with *K* yields that $n=\dim _{K}(K \otimes N)$.

#### Proof

The vector space $K\otimes N$ is contained in the finite-dimensional vector space $K \otimes M$. Hence there exist $v_1,\ldots ,v_n \in N$ such that $1 \otimes v_1, \ldots , 1 \otimes v_n$ is a basis of $K \otimes N$, and $v_{n+1}, \ldots , v_m \in M$ such that $1 \otimes v_{n+1}, \ldots , 1 \otimes v_m$ is a basis of a complement of $K \otimes N$ in $K \otimes M$. We claim that both statements hold with *K* replaced by *R*[1/*r*] for some nonzero *r*.

To see this, extend $v_1,\ldots ,v_m$ with $v_{m+1},\ldots ,v_l$ to a generating set of the *R*-module *M*. Then for each $j=m+1,\ldots ,l$ we have, in $K \otimes M$,

$$\begin{aligned} 1 \otimes v_j=\sum _{i=1}^m c_{ij} \otimes v_i \end{aligned}$$

for certain coefficients $c_{ij} \in K$. This identity means that there exists a non-zero element $r\in R$ and suitable coefficients $c_{ij}$’s in *R* such that

$$\begin{aligned} 1 \otimes v_j = \sum _{i=1}^m (c'_{ij}/r) \otimes v_i \end{aligned}$$

holds in $R[1/r] \otimes M$. Hence $R[1/r] \otimes M$ is generated by $1 \otimes v_1, \ldots , 1 \otimes v_m$, and these elements do not have any nontrivial linear relation over *R*[1/*r*] since their images in $K \otimes M$ do not satisfy any such relation over *K*. It follows that $R[1/r] \otimes M$ is free with basis $1 \otimes v_1,\ldots ,1 \otimes v_m$. Furthermore, $R[1/r] \otimes N$ contains the *R*[1/*r*]-module spanned by $1 \otimes v_1,\ldots ,1 \otimes v_n$; and conversely, if $v \in R[1/r] \otimes M$ is an element of $R[1/r] \otimes N$, then it cannot have a nonzero coefficient on any of the last $m-n$ basis elements, because in $K \otimes M$ the image of *v* is a linear combination of the first *m* basis elements and the basis elements do not satisfy any linear relation there. Hence $R[1/r] \otimes N \subseteq R[1/r] \otimes M$ is free with basis $1 \otimes v_1,\ldots ,1 \otimes v_n$. $\square$

## Polynomial laws and the coordinate ring of a module

### Polynomial laws

We follow [22, Chapter 1]. Let *M*, *N* be *R*-modules. Denote by ${{\,\mathrm{\mathbf {Alg}_R}\,}}$ the category of *R*-algebras.

#### Definition 9

A *polynomial law* $\varphi :M \rightarrow N$ is a collection of maps

$$\begin{aligned} (\varphi _A:A \otimes M \rightarrow A \otimes N)_{A\in {{\,\mathrm{\mathbf {Alg}_R}\,}}} \end{aligned}$$

such that for every *R*-algebra homomorphism $\alpha :A \rightarrow B$ the following diagram commutes:

#### Example 10

Suppose that *M* and *N* are the free modules $R^2$ and *R*, respectively, so that $A \otimes M$ and $A \otimes N$ are canonically identified with $A^2$ and *A*. Then the collection $(\varphi _A)_A$ defined by $\varphi _A(x,y)=xy+y^2$ for $x,y \in A$ is a polynomial law $M \rightarrow N$, and indeed one that is *homogeneous* of degree 2 in the sense of Definition 13 below.

More generally, the name polynomial law derives from the following fact.

#### Lemma 11

Consider two *R*-modules *M* and *N*. Suppose that *M* is finitely generated and let $\{v_1,\ldots ,v_n\}$ be a set of generators. Let $\varphi :M\rightarrow N$ be a polynomial law. Then $\varphi$ is completely determined by the element:

$$\begin{aligned} \iota (\varphi ):=\varphi _{R[x_1,\ldots ,x_n]}(x_1 \otimes v_1 + \cdots + x_n \otimes v_n) \in R[x_1,\ldots ,x_n] \otimes N. \end{aligned}$$

This gives an injective map $\iota$ from the collection of polynomial laws from *M* to *N* to the module $R[x_1,\ldots ,x_n] \otimes N$. In the case where *M* is free with basis $v_1,\ldots ,v_n$, this injective map is a bijection.

#### Proof

Let *A* be an *R*-algebra, let $a_1,\ldots ,a_n\in A$ be elements and let $\alpha :R[x_1,\ldots ,x_n]\rightarrow A$ be the *R*-algebra homomorphism sending $x_i\mapsto a_i$. Then the diagram associated to $\alpha$ shows that $\varphi _A(a_1\otimes v_1+\cdots +a_n\otimes v_n)=(\alpha \otimes {{\,\mathrm{id}\,}}_N)\iota (\varphi )$ and hence $\iota$ is injective. If *M* is free with basis $v_1,\ldots ,v_n$, then $\varphi _A(a_1\otimes v_1+\cdots +a_n\otimes v_n)=\sum _jf_j(a_1,\ldots ,a_n)\otimes w_j$ defines a polynomial law $\varphi :M\rightarrow N$ for every $\sum _j f_j\otimes w_j\in R[x_1,\ldots ,x_n]\otimes N$. $\square$

#### Example 12

If *R* is an infinite field, then a polynomial law $\varphi$ from $M=R^n$ to $N=R^m$ is in fact uniquely determined by $\varphi _R$, which is required to be a polynomial map, i.e., a map all of whose *m* coordinate functions are polynomials in the *n* coordinates on *M*. So then the set of polynomial laws from *M* to *N* is precisely the set of polynomial maps from the vector space *M* to the vector space *N*.

For a general ring *R*, we denote by ${{\,\mathrm{{\mathbb A}}\,}}_R^n$ the affine scheme ${{\,\mathrm{Spec}\,}}(R[x_1, \ldots , x_n])$. The set of polynomial laws from $R^n$ to $R^m$ is the set of morphisms ${{\,\mathrm{{\mathbb A}}\,}}_R^n \rightarrow {{\,\mathrm{{\mathbb A}}\,}}_R^m$ defined over *R*. Of course, such a morphism need not be determined by its map $\varphi _R:R^n \rightarrow R^m$, but it *is* determined by the maps $\varphi _A:A^n \rightarrow A^m$ for all *R*-algebras *A*. This motivates the definition of polynomial laws.

#### Definition 13

A polynomial law $\varphi :M \rightarrow N$ is *homogeneous of degree d* if for each *R*-algebra *A* and all $a \in A, m \in A \otimes M$, we have $\varphi _A(am)=a^d \varphi _A(m)$.

Writing $R[x_1,\ldots ,x_n]_d$ for the set of homogeneous polynomials of degree *d*, we see that the injection from Lemma 11 maps a homogeneous polynomial law $M \rightarrow N$ of degree *d* to an element of $R[x_1,\ldots ,x_n]_d \otimes N$.

#### Proposition 14

Let $M_1,\ldots ,M_d,N$ be *R*-modules and let $\varphi :M_1\times \cdots \times M_d\rightarrow N$ be a multilinear map. Then $\varphi$ extends to a homogeneous polynomial law of degree *d* (also denoted $\varphi$). After identifying $A\otimes (M_1\times \cdots \times M_d)\cong A\otimes M_1\times \cdots \times A\otimes M_d$, we have

$$\begin{aligned} \varphi _A\left( \sum _{i_1}a_{i_1}\otimes m_{i_1},\ldots ,\sum _{i_d}a_{i_d}\otimes m_{i_d}\right) =\sum _{i_1,\ldots ,i_d}a_{i_1}\cdots a_{i_d}\otimes \varphi (m_{i_1},\ldots ,m_{i_d}) \end{aligned}$$

for all *R*-algebras *A*, $a_{i_1},\ldots ,a_{i_d}\in A$ and $m_{i_1}\in M_1,\ldots ,m_{i_d}\in M_d$.

#### Proof

The maps $\varphi _A$ are well-defined as the maps $A^d\times M_1\times \cdots \times M_d\rightarrow A \otimes N$ sending $(a_1,\ldots ,a_d,m_1,\ldots ,m_d)\mapsto a_1\cdots a_d\varphi (m_1\cdots m_d)$ are multilinear. The collection $(\varphi _A)_A$ is a homogeneous polynomial law of degree *d* and $\varphi _R=\varphi$. $\square$

#### Remark 15

Composition of *R*-module homomorphisms is a bilinear map. By the proposition, we can thus view this operation as a polynomial law.

A homogeneous polynomial law $\varphi :M \rightarrow N$ of degree 0 is the same thing as an element of *N* (namely, the element $\varphi _R(0)$, which equals $\varphi _A(m)$ for any *R*-algebra *A* and any element $m \in A \otimes M$); we call these polynomial laws *constant*. A homogeneous polynomial law $M \rightarrow N$ of degree 1 is the extension of an *R*-module homomorphism $M \rightarrow N$ as in the proposition above (namely, the map $\varphi _R:M \rightarrow N$, which in this case is *R*-linear and uniquely determines $\varphi _A$ for all $A \in {{\,\mathrm{\mathbf {Alg}_R}\,}}$); we call these polynomial laws *linear*.

The following proposition says that, in many ways, polynomial laws behave like ordinary polynomial maps between vector spaces. For proofs we refer to [22].

#### Proposition 16

Let $\varphi ,\psi :M \rightarrow N$, $\gamma :N \rightarrow O$ be polynomial laws between *R*-modules.

1. The collection $\varphi +\psi :=(\varphi _A+\psi _A)_A$ is a polynomial law $M \rightarrow N$, homogeneous of degree *d* if $\varphi ,\psi$ are.
2. We have $\varphi =\sum _{d=0}^\infty \varphi _d$ for unique polynomial laws $\varphi _d:M \rightarrow N$ of degree *d*, where for each *R*-algebra *A* and each $m \in A \otimes M$ we have $\varphi _{d,A}(m)=0$ for all but finitely many *d*’s ($\varphi _d$ is called the *homogeneous component* of $\varphi$ of degree *d*); moreover, if *M* is finitely generated, then only finitely many of the $\varphi _d$ are nonzero.
3. The collection $\gamma \circ \varphi :=(\gamma _A \circ \varphi _A)_A$ is a polynomial law $M \rightarrow O$, homogeneous of degree $d \cdot e$ if $\varphi ,\psi$ are homogeneous of degrees *d*, *e*, respectively.
4. If $N=R$, then $\varphi \cdot \psi =(m \mapsto \varphi _A(m) \psi _A(m))_A$ is a polynomial law $M \rightarrow R$, homogeneous of degree $d+e$ if $\varphi ,\psi$ are homogeneous of degrees *d*, *e*, respectively.

#### Proposition 17

Let $\varphi :M\oplus M'\rightarrow N$ be a polynomial law between *R*-modules. Then $\varphi$ has a unique decomposition $\varphi =\sum _{i,j=0}^\infty \varphi _{(i,j)}$ such that $\varphi _{(i,j)}:M\oplus M'\rightarrow N$ is a bihomogeneous polynomial law of degree (*i*, *j*), i.e., after identifying $A\otimes (M\oplus M')\cong A\otimes M\oplus A\otimes M'$, we have $\varphi _{(i,j),A}(am,bm')=a^ib^j\varphi _{(i,j),A}(m,m')$ for all *R*-algebras *A*, $a,b\in A$, $m\in A\otimes M$ and $m'\in A\otimes M'$. Moreover, if $\varphi$ is homogeneous of degree *d*, then $\varphi _{(i,j)}=0$ for all $i+j\ne d$.

#### Proof

Suppose that such a decomposition exists and let *A* be an *R*-algebra. Then we have

$$\begin{aligned} \varphi _{A[s,t]}(sm,tm')= & {} \sum _{i,j}\varphi _{(i,j),A[s,t]}(sm,tm')\\= & {} \sum _{i,j}s^it^j\varphi _{(i,j),A}(m,m')\in \bigoplus _{i,j=0}^\infty s^it^jA\otimes N \end{aligned}$$

for all $m\in A\otimes M$ and $m'\in A\otimes M'$. This shows that the $\varphi _{(i,j)}$ are unique. If $\varphi$ is homogeneous of degree *d*, setting $s=t$, we see that $\varphi =\sum _{i+j=d}\varphi _{(i,j)}$ and hence $\varphi _{(i,j)}=0$ for $i+j\ne d$. What remains to show the existence of the decomposition. In fact, defining $\varphi _{(i,j),A}(m,m')$ to be the coefficient of $s^it^j$ in $\varphi _{A[s,t]}(sm,tm')$, it is easy to show that the $\varphi _{(i,j)}$ are bihomogeneous polynomial laws of degree (*i*, *j*) adding up to $\varphi$. $\square$

The class of *R*-modules, in addition to its structure of Abelian category with *R*-module homomorphisms as morphisms, has the structure of a (non-Abelian) category with polynomial laws as morphisms. Both structures will be important to us, but we reserve the notation ${{\,\mathrm{\mathbf {Mod}_R}\,}}$ for the category in which the morphisms are *R*-module homomorphisms (i.e., homogeneous polynomial laws of degree 1).

#### Definition 18

(*Base change*). If *B* is an *R*-algebra, then the tensor product functor ${{\,\mathrm{\mathbf {Mod}_R}\,}}\rightarrow {{\,\mathrm{\mathbf {Mod}_B}\,}}$, which sends *linear* polynomial laws over *R* to linear polynomial laws over *B*, can be extended to a functor from the category of *R*-modules with polynomial laws over *R* to the category of *B*-modules with polynomial laws over *B*: on objects, the functor is just $M \mapsto B \otimes M$, and a polynomial law $(\varphi _A)_{A \in {{\,\mathrm{\mathbf {Alg}_R}\,}}}:M \rightarrow N$ is mapped to $(\varphi _A)_{A \in {{\,\mathrm{\mathbf {Alg}_B}\,}}}$ where, for a *B*-algebra *A*, the map $\varphi _A$ is interpreted as a map $A \otimes _B (B \otimes _R M) \cong A \otimes _R M \rightarrow A \otimes _R N \cong A \otimes _B (B \otimes _R N)$.

### The coordinate ring of a module

Let *M* be a finitely generated *R*-module.

#### Definition 19

We write *R*[*M*] for the set of polynomial laws $M\rightarrow R$ and $R[M]_d\subseteq R[M]$ for the subset of homogeneous polynomial laws of degree *d*. The addition and multiplication from Proposition 16, the grading from Definition 13 and the identification $R[M]_0=R$ give $R[M]=\bigoplus _{d=0}^\infty R[M]_d$ the structure of a ${{\,\mathrm{{\mathbb Z}}\,}}_{\ge 0}$-graded commutative *R*-algebra. We call this *R*-algebra the *coordinate ring* of *M*.

#### Remark 20

In [22, Chapitre III], various algebras associated to an *R*-module *M* are introduced, but they are different from our *R*-algebra *R*[*M*]. One important difference is that for us, the elements of *M* play the role of geometric objects, whereas there, the algebras consist of elements in divided or symmetric powers of *M*.

As usual with coordinate rings, the association $M \mapsto R[M]$ is a contravariant functor from the category of *R*-modules with polynomial laws to the category of *R*-algebras: a polynomial law $\varphi :M \rightarrow N$ has a pull-back map $\varphi ^*:R[N] \rightarrow R[M]$ sending $f \mapsto f \circ \varphi$. If $\varphi$ is linear, then $\varphi ^*$ is a graded homomorphism.

If *M* is generated by $v_1,\ldots ,v_n$, then the injection $\iota :R[M]\rightarrow R[x_1,\ldots ,x_n]$ of Lemma 11 is a graded ring homomorphism. The following lemma says precisely which subalgebra its image is.

#### Lemma 21

Let $\psi :N \rightarrow M$ be a surjective *R*-module homomorphism. Then the map $\psi ^*$ is a graded isomorphism from *R*[*M*] to the graded *R*-subalgebra of *R*[*N*] whose degree-*d* part equals

$$\begin{aligned} \{f \in R[N]_d \mid \forall u \in \ker (\psi ): f \circ t_u = f \} \end{aligned}$$

where $t_u:N \rightarrow N$ (called *translation by u*) is the affine-linear polynomial law $v \mapsto v+u$.

#### Proof

Let $g\in R[M]_d$ and write $f=\psi ^*(g)=g\circ \psi$. To see that $\psi ^*$ is injective, note that $f_A=g_A\circ ({{\,\mathrm{id}\,}}_A\otimes \,\psi )$ for all *R*-algebras *A*. So if $f_A=0$, then $g_A=0$ as ${{\,\mathrm{id}\,}}_A\otimes \,\psi$ is surjective. To see that the image is contained in the subalgebra, it is enough to note that $\psi _A={{\,\mathrm{id}\,}}_A\otimes \,\psi$ and $t_{u,A}(m)=m+1\otimes u$ and so $\psi \circ t_u=\psi$ as polynomial laws. Now, let $f \in R[N]_d$ be a polynomial law such that $f\circ t_u = f$ for all $u\in \ker (\psi )$. It remains to show that $f=g\circ \psi$ for some $g\in R[M]_d$. As ${{\,\mathrm{id}\,}}_A\otimes \,\psi$ is surjective, we set $g_A(m):=f_A(n)$ for any $n\in A\otimes N$ mapping to *m*. To do this, we need to show that $f_A(n)=f_A(n')$ whenever $n-n'\in \ker ({{\,\mathrm{id}\,}}_A\otimes \,\psi )$. Since the functor $A \otimes -$ from *R*-modules to *A*-modules is right-exact, we have $\ker ({{\,\mathrm{id}\,}}_A\otimes \,\psi )=A \otimes \ker (\psi )$. Take $h=f\circ ((n,n')\mapsto n+n')$. Then we see that

$$\begin{aligned} h_A(n,1\otimes u)=f_A(n+1\otimes u)=(f\circ t_u)_A(n)=f_A(n)=h_A(n,0) \end{aligned}$$

for all *R*-algebras *A*, $n\in A\otimes N$ and $u\in \ker (\psi )$. It follows that $h_{(i,j),A}(n,1\otimes u)=0$ whenever $j>0$. And, we have $h_{(d,0),A}(n,n')=f_A(n)$. So

$$\begin{aligned} f_A(n+a\otimes u)= & {} h_A(n,a\otimes u)\\= & {} h_{(d,0),A}(n,a\otimes u)+\sum _{i=1}^dh_{(d-i,i),A}(b,a\otimes u)\\= & {} f_A(n)+\sum _{i=1}^da^ih_{(d-i,i),A}(b,1\otimes u)\\= & {} f_A(n) \end{aligned}$$

for all $n\in A\otimes N$, $a\in A$ and $u\in \ker (\psi )$. So if $n-n'\in \ker ({{\,\mathrm{id}\,}}_A\otimes \,\psi )$, then $f_A(n)=f_A(n')$. This shows $g_A$ is well-defined. It is straightforward to check that $g=(g_A)_A$ is a homogeneous polynomial law of degree *d*. $\square$

#### Example 22

When *R* is an infinite field and both *M* and *N* are finite-dimensional vector spaces over *R*, *R*[*M*] is just the subring of *R*[*N*] consisting of all polynomials that are constant on fibres of the projection $N \rightarrow M$.

The following example shows that, even when *R* is Noetherian and *M* is finitely generated, *R*[*M*] need not be Noetherian.

#### Example 23

Let $R:=K[t]/(t^2)$ where *K* is a field of characteristic zero, and let $M:=K[t]/(t)$. Then $M=R/(t)$ is an *R*-module generated by a single element $v:=1+(t)$ and *R*[*M*] is the subring of *R*[*x*] spanned by all homogeneous polynomials $f=c x^d$ such that $f(x+at)=f(x)$ for all $a \in K$. Now $c(x+at)^d=cx^d + cdatx^{d-1}$ and hence we need that $c \in (t)$ whenever $d \ge 1$. Hence *R*[*M*] is the vector space over *K* spanned by $1,t,tx,tx^2,\ldots$ with the multiplication $(t^ix)(t^ix)=0$. Observe that *R*[*M*] is not Noetherian, since the ideal $\mathrm {span}\{t,tx,tx^2,\ldots \}$ is not finitely generated. On the other hand, the quotient $R[M]^{{{\,\mathrm{red}\,}}}$ of *R*[*M*] by its ideal of nilpotent elements is *K*.

However, we will see later that if ${{\,\mathrm{Spec}\,}}(R)$ is Noetherian and *M* is finitely generated, then a certain topological space ${{\,\mathrm{{\mathbb A}}\,}}_M$ defined using *R*[*M*] is also Noetherian. In Example 23, this is a consequence of the fact that ${{\,\mathrm{Spec}\,}}(R[M])={{\,\mathrm{Spec}\,}}(K)$ is Noetherian. See also Remark 41.

#### Example 24

Now consider a field *K* of characteristic 2 and set $R:=K[t]/(t^2)$. The same computation as above shows that $cx^i$ with *odd* *i* can only be in $R[M] \subseteq R[x]$ if *c* is in (*t*). But for *even* *i*, $cx^i$ is in *R*[*M*] regardless of $c \in R$. Hence *R*[*M*] is the *K*-vector space with basis

$$\begin{aligned} 1,t,tx,x^2,tx^2,tx^3,x^4,tx^4,\ldots \end{aligned}$$

and $R[M]^{{{\,\mathrm{red}\,}}} \cong K[x^2]$ as a graded algebra.

If *B* is an *R*-algebra, then the base change functor from Definition 18 sends polynomial laws $M \rightarrow R$ to polynomial laws $B \otimes M \rightarrow B$. This yields an *R*-algebra homomorphism $R[M] \rightarrow B[B\otimes M]$ and hence a *B*-algebra homomorphism $B \otimes R[M] \rightarrow B[B \otimes M]$. The following example shows that this needs not be an isomorphism.

#### Example 25

Let $R={{\,\mathrm{{\mathbb Z}}\,}}$ and $M={{\,\mathrm{{\mathbb Z}}\,}}/2{{\,\mathrm{{\mathbb Z}}\,}}$, generated by a single element $v=1+2{{\,\mathrm{{\mathbb Z}}\,}}$. Then by Lemma 21, *R*[*M*] is the subring of *R*[*x*] spanned by all homogeneous univariate polynomials *f* such that $f(x+2a)=f(x)$ for all $a \in {{\,\mathrm{{\mathbb Z}}\,}}$. Only the constant polynomials have that property, so $R[M]=R$. Now take the ${{\,\mathrm{{\mathbb Z}}\,}}$-algebra $B={{\,\mathrm{{\mathbb Z}}\,}}/2{{\,\mathrm{{\mathbb Z}}\,}}=:{{\,\mathrm{{\mathbb F}}\,}}_2$, which is a field, and $B \otimes M$ is the one-dimensional vector space over that field, so $B[B \otimes M] \cong {{\,\mathrm{{\mathbb F}}\,}}_2[x]$.

However, when *B* is a localisation of a domain *R*, then the map *is* an isomorphism:

#### Proposition 26

Suppose that *R* is a domain. Let *M* be a finitely generated *R*-module and let *S* be a multiplicative subset of *R* not containing 0. Set $R':=S^{-1} R$. Then

$$\begin{aligned} R'\otimes R[M] \cong S^{-1} R[M] \cong R'[R' \otimes M]\cong R'[S^{-1}M]. \end{aligned}$$

#### Proof

The first and last isomorphisms are standard. For the middle isomorphism, we choose generators $m_1,\ldots ,m_n$ of *M* and embed *R*[*M*] as a graded *R*-subalgebra *A* of $R[x_1,\ldots ,x_n]$. Since localisation is exact, $S^{-1}R[M]$ is then isomorphic to the $R'$-algebra $S^{-1} A \subseteq R'[x_1,\ldots ,x_n]$. On the other hand, using the generators $1 \otimes m_1,\ldots ,1 \otimes m_n$, the $R'$-algebra $R'[R' \otimes M]$ also embeds as a graded $R'$-subalgebra *B* of $R'[x_1,\ldots ,x_n]$. The canonical map $R' \otimes R[M] \rightarrow R'[R' \otimes M]$ translates into an inclusion $S^{-1} A \subseteq B$, so it remains to show that $B \subseteq S^{-1} A$. For this, let *O* be the kernel of the *R*-module homomorphism $R^n \rightarrow M$ given by the generators $m_1,\ldots ,m_n$. Again since localisation is exact, $S^{-1}O \cong R' \otimes O$ is the kernel of the corresponding $R'$-module homomorphism $(R')^n \rightarrow R' \otimes M$. Let $f \in B$ and let $s \in S$ be such that $g:=sf \in R[x_1,\ldots ,x_n]$. Then, since $f \in B$, one has that $f \circ t_u = f$ for all $u \in S^{-1}O \subseteq (R')^n$, by Lemma 21 applied to the $R'$-module $R' \otimes M$. In particular, the multiplication by *s* gives $g \circ t_u = g$ over $R'$ for all $u \in O \subseteq R^n$. Since *R* is a domain, the same holds over *R* and hence $g \in A$, again by Lemma 21 but now applied to the *R*-module *M*. Hence $f=s^{-1} g \in S^{-1} A$, as desired. $\square$

Like in ordinary algebraic geometry, the coordinate ring of a direct sum is the tensor product of the coordinate rings.

#### Proposition 27

Let *M*, *N* be finitely generated *R*-modules. Then

$$\begin{aligned} R[M \oplus N] \cong R[M]\otimes R[N]. \end{aligned}$$

#### Proof

Elements of *R*[*M*] and *R*[*N*] induce elements of $R[M\oplus N]$ via composition with the projections $M\oplus N\rightarrow M$ and $M\oplus N\rightarrow N$, respectively. The product of such induced polynomial laws $M\oplus N\rightarrow R$ gives a bilinear map $R[M]\times R[N]\rightarrow R[M\oplus N]$. This induces an *R*-linear map $R[M]\otimes R[N]\rightarrow R[M\oplus N]$, which is in fact a homomorphism of *R*-algebras. Denote by $R[M\oplus N]_{(d,e)}$ the *R*-submodule of $R[M\oplus N]$ consisting of all bihomogeneous polynomial laws of degree (*d*, *e*). It suffices to show that $R[M\oplus N]_{(d,e)}\cong R[M]_d\otimes R[N]_e$. To see this, first suppose that *M*, *N* are free. In this case, we get $R[x_1,\ldots ,x_n,y_1,\ldots ,y_m]_{(d,e)}\cong R[x_1,\ldots ,x_n]_d\otimes R[y_1,\ldots ,y_m]_e$ when $x_i,y_j$ have degrees (1, 0), (0, 1), respectively. In general, let $\varphi :M'\rightarrow M$ and $\psi :N'\rightarrow N$ be surjective *R*-linear maps from finitely generated free *R*-modules. Then we see that

$$\begin{aligned}&\{f \in R[M'\oplus N']_{(d,e)} \mid \forall u_1 \in \ker (\varphi )\forall u_1\in \ker (\psi ) : f \circ t_{(u_1,u_2)} = f \}\\&\quad \cong \{f \in R[M']_d \mid \forall u_1 \in \ker (\varphi ): f \circ t_{u_1} = f \}\otimes \{g \in R[N']_e \mid \forall u_1\in \ker (\psi ) :\\&\quad g \circ t_{u_2} = g\} \end{aligned}$$

and hence $$R[M\oplus N]_{(d,e)}\cong R[M]_d\otimes R[N]_e$$. $$\square $$

Example 23 shows that the coordinate ring of a module is quite a subtle notion. However, we will see that in the proof of our Theorem 2, by a localisation we can always pass to a case where the module *M* is free. In that case, by Lemma 21, *R*[*M*] is just a polynomial ring over *R*.

## The topological space ${{\,\mathrm{{\mathbb A}}\,}}_M$

### The space ${{\,\mathrm{{\mathbb A}}\,}}_M$

We now construct the topological space ${{\,\mathrm{{\mathbb A}}\,}}_M$ for *M* a finitely generated *R*-module. To be precise, ${{\,\mathrm{{\mathbb A}}\,}}_M$ is a topological space over the category ${{\,\mathrm{\mathbf {Dom}_R}\,}}$ of *R*-domains with *R*-algebra monomorphisms, in the sense of the following definition.

#### Definition 28

Let $F:\mathbf {C}\rightarrow \mathbf {D}$ be a functor and suppose that the objects of $\mathbf {D}$ are sets and the morphisms are maps (i.e, we have a forgetful functor ${{\,\mathrm{Forget}\,}}:\mathbf {D}\rightarrow {{\,\mathrm{\mathbf {Set}}\,}}$). An *element* of *F* is an element of *F*(*C*) for some $C\in \mathbf {C}$. A *subset* of *F* is a subfunctor of ${{\,\mathrm{Forget}\,}}\circ F$, i.e., a rule *X* that assigns to each $C\in \mathbf {C}$ a subset $X(C)\subseteq F(C)$ in such a manner that $F_{C,D}(\varphi )$ maps *X*(*C*) into *X*(*D*) for all morphisms $\varphi :C\rightarrow D$. A *topological space over* $\mathbf {C}$ is a pair $(F,\mathcal {T})$ where *F* is a functor as above and $\mathcal {T}$ is a collection of subsets of *F* including the subsets $\emptyset ,F$ that is closed under taking arbitrary intersections and finite unions.

#### Remark 29

We note that all definitions that can be stated in terms of elements and (closed) subsets of a topological space carry over to topological spaces over $\mathbf {C}$. We also note that a topological space $(F,\mathcal {T})$ gives rise to a functor from $\mathbf {C}$ to the category of topological spaces, which sends *C* to the set *F*(*C*) with the collection $\{X(C) \mid X \in \mathcal {T}\}$ of closed subsets. Clearly, not every functor from $\mathbf {C}$ to the category of topological spaces arises in this manner.

In what follows, we use the term *“injections*" to refer to *R*-algebra monomorphisms.

#### Definition 30

Define ${{\,\mathrm{{\mathbb A}}\,}}_M$ to be the rule assigning to each $D \in {{\,\mathrm{\mathbf {Dom}_R}\,}}$ the set $D \otimes M$. A subset of ${{\,\mathrm{{\mathbb A}}\,}}_M$ is a rule *X* that assigns to each $D \in {{\,\mathrm{\mathbf {Dom}_R}\,}}$ a subset *X*(*D*) of $D \otimes M$ in such a manner that $\iota \otimes {{\,\mathrm{id}\,}}_M$ maps *X*(*D*) into *X*(*E*) for all injections $\iota :D\rightarrow E$. For every subset $S \subseteq R[M]$, the rule ${{\,\mathrm{\mathcal {V}}\,}}(S)$ assigning

$$\begin{aligned} D\mapsto {{\,\mathrm{\mathcal {V}}\,}}(S)(D):=\{m \in D \otimes M \mid \forall f \in S:f_D(m)=0\} \end{aligned}$$

is a subset of ${{\,\mathrm{{\mathbb A}}\,}}_M$. We say that $X\subseteq {{\,\mathrm{{\mathbb A}}\,}}_M$ is *closed* if $X={{\,\mathrm{\mathcal {V}}\,}}(S)$ for some $S\subseteq R[M]$. This collection of closed sets makes ${{\,\mathrm{{\mathbb A}}\,}}_M$ into a topological space over ${{\,\mathrm{\mathbf {Dom}_R}\,}}$ in the sense of Definition 28.

#### Remark 31

If *D* is an *R*-domain, then we can make $D \otimes M$ into an topological space by defining the closed subsets to be ${{\,\mathrm{\mathcal {V}}\,}}(S)(D)$ for $S\subseteq R[M]$; we will call this the Zariski topology (over *R*) on $D \otimes M$. To see that these sets are preserved under finite unions, one uses ${{\,\mathrm{\mathcal {V}}\,}}(S)(D) \cup {{\,\mathrm{\mathcal {V}}\,}}(T)(D)= {{\,\mathrm{\mathcal {V}}\,}}(S \cdot T)(D)$, which holds since *D* is a domain. For any *R*-algebra homomorphism $D \rightarrow E$ between *R*-domains (not necessarily injective), the induced map $D \otimes M \rightarrow E \otimes M$ sends ${{\,\mathrm{\mathcal {V}}\,}}(S)(D)$ into ${{\,\mathrm{\mathcal {V}}\,}}(S)(E)$. Furthermore, if $D \rightarrow E$ is injective, then that induced map is continuous with respect to the topologies on $D \otimes M$ and $E \otimes M$. So ${{\,\mathrm{{\mathbb A}}\,}}_M$ induces a functor from ${{\,\mathrm{\mathbf {Dom}_R}\,}}$ to ${{\,\mathrm{\mathbf {Top}}\,}}$ and the ${{\,\mathrm{\mathcal {V}}\,}}(S)$ are closed subfunctors. In this paper, however, we will not consider closed subsets of $D\otimes M$ on their own.

#### Remark 32

We think of ${{\,\mathrm{{\mathbb A}}\,}}_M$ as the “affine space” corresponding to *M*. Note that in the definition of closed subsets of ${{\,\mathrm{{\mathbb A}}\,}}_M$ we require *S* to be independent of *D*, i.e., not every rule assigning to $D\in {{\,\mathrm{\mathbf {Dom}_R}\,}}$ a subset of the form ${{\,\mathrm{\mathcal {V}}\,}}(S)(D)$ is a closed subset of ${{\,\mathrm{{\mathbb A}}\,}}_M$. To see that this is desirable, consider $R={{\,\mathrm{{\mathbb Z}}\,}}$, $M=R$ and let $X_n$ be the rule such that $X_n(D)=\{0\}={{\,\mathrm{\mathcal {V}}\,}}(\{x\})(D)$ when $0<{{\,\mathrm{char}\,}}D\le n$ and $X_n(D)=D={{\,\mathrm{\mathcal {V}}\,}}(\emptyset )(D)$ otherwise. Then $X_1\supseteq X_2\supseteq X_3\supseteq \cdots$ is a descending chain of rules and $X_{p-1}({{\,\mathrm{{\mathbb F}}\,}}_p)={{\,\mathrm{{\mathbb F}}\,}}_p\ne \{0\}=X_p({{\,\mathrm{{\mathbb F}}\,}}_p)$ for every prime number $p>0$.

#### Definition 33

(*Base change*). If *B* is an *R*-algebra, and *D* is a *B*-domain, then $D \otimes M \cong D \otimes _B (B \otimes M)$ also carries a Zariski topology over *B*, coming from closed sets defined by subsets of $B[B \otimes M]$. This refines the Zariski topology on $D \otimes M$ over *R*. If *X* is a closed subset of ${{\,\mathrm{{\mathbb A}}\,}}_M$, then we write $X_B$ for the closed subset of ${{\,\mathrm{{\mathbb A}}\,}}_{B \otimes M}$ that maps a *B*-domain *D* to *X*(*D*).

Let *X* be a subset of ${{\,\mathrm{{\mathbb A}}\,}}_M$. Then we define the *ideal* of *X* to be

$$\begin{aligned} {{\,\mathrm{\mathcal {I}}\,}}_X:=\{ f\in R[M]\mid \forall D\in {{\,\mathrm{\mathbf {Dom}_R}\,}}\forall x\in X(D): f_D(x)=0\}. \end{aligned}$$

As $f_D$ maps elements into a domain, we see that ${{\,\mathrm{\mathcal {I}}\,}}_X$ is a radical ideal of *R*[*M*]. We define the *closure* of *X* in ${{\,\mathrm{{\mathbb A}}\,}}_M$ to be the closed subset $\overline{X}:={{\,\mathrm{\mathcal {V}}\,}}({{\,\mathrm{\mathcal {I}}\,}}_X)$ of ${{\,\mathrm{{\mathbb A}}\,}}_M$.

Let $\varphi :M\rightarrow N$ be a polynomial law between finitely generated *R*-modules. Then the maps $(\varphi _D)_{D\in {{\,\mathrm{\mathbf {Dom}_R}\,}}}$ define a continuous map ${{\,\mathrm{{\mathbb A}}\,}}_M\rightarrow {{\,\mathrm{{\mathbb A}}\,}}_N$, i.e., for every injection $\iota :D\rightarrow E$, the diagramcommutes, so $\varphi (X)=(D\mapsto \varphi _D(X(D)))$ is a subset of ${{\,\mathrm{{\mathbb A}}\,}}_N$ for each subset *X* of ${{\,\mathrm{{\mathbb A}}\,}}_M$, and for every subset $S\subseteq R[N]$, the subset

$$\begin{aligned} \varphi ^{-1}({{\,\mathrm{\mathcal {V}}\,}}(S))=(D\mapsto \varphi _D^{-1}({{\,\mathrm{\mathcal {V}}\,}}(S)(D)))_D \end{aligned}$$

of ${{\,\mathrm{{\mathbb A}}\,}}_M$ is closed (as $\varphi _D^{-1}({{\,\mathrm{\mathcal {V}}\,}}(S)(D))={{\,\mathrm{\mathcal {V}}\,}}(\varphi ^*S)(D)$ holds). As usual, we have

$$\begin{aligned} \varphi (\overline{X})\subseteq \overline{\varphi (X)} \end{aligned}$$

for all subsets *X* of ${{\,\mathrm{{\mathbb A}}\,}}_M$.

When *M* is free and finitely generated, we have the usual correspondence between closed subsets and radical ideals.

#### Proposition 34

Let *M* be a finitely generated free *R*-module of rank *n*. Then the rule sending an element $x\in D\otimes M$ of ${{\,\mathrm{{\mathbb A}}\,}}_M$ to $\mathfrak {q}_x:=\{f\in R[M]\mid f_D(x)=0\}\in {{\,\mathrm{{\mathbb A}}\,}}^n_R:={{\,\mathrm{Spec}\,}}(R[M])$ is surjective and maps closed subsets of ${{\,\mathrm{{\mathbb A}}\,}}_M$ to closed subsets of ${{\,\mathrm{{\mathbb A}}\,}}^n_R$. Moreover, that map from closed subsets of ${{\,\mathrm{{\mathbb A}}\,}}_M$ to closed subsets of ${{\,\mathrm{{\mathbb A}}\,}}^n_R$ is a bijection. In particular, we have ${{\,\mathrm{\mathcal {I}}\,}}_{{{\,\mathrm{\mathcal {V}}\,}}(S)}={{\,\mathrm{rad}\,}}(S)$ for any subset $S\subseteq R[M]$.

#### Proof

Note that for every *R*-domain *D* and element $x\in D\otimes M$, the set $\mathfrak {q}_x\subseteq R[M]$ is a prime ideal. Let $\mathfrak {q}\subseteq R[M]=R[x_1,\ldots ,x_n]$ be a prime ideal. Then we have $\mathfrak {q}=\mathfrak {q}_x$ for $x=(x_1+\mathfrak {q},\ldots ,x_n+\mathfrak {q})\in (R[M]/\mathfrak {q})\otimes M$. Next, let $S\subseteq R[M]$ be a set. Then we see that $\{\mathfrak {q}_x\mid x\in {{\,\mathrm{\mathcal {V}}\,}}(S)(D), D\in {{\,\mathrm{\mathbf {Dom}_R}\,}}\}=\{\mathfrak {q}\in {{\,\mathrm{Spec}\,}}(R[M])\mid \mathfrak {q}\supseteq S\}$. So closed subsets of ${{\,\mathrm{{\mathbb A}}\,}}_M$ are mapped to closed subsets of ${{\,\mathrm{{\mathbb A}}\,}}^n_R$. Clearly, every closed subset arises from a closed subset of ${{\,\mathrm{{\mathbb A}}\,}}_M$. To see that this map is injective, we note that

$$\begin{aligned} {{\,\mathrm{\mathcal {I}}\,}}_{{{\,\mathrm{\mathcal {V}}\,}}(S)}=\bigcap _{\begin{array}{c} x\in {{\,\mathrm{\mathcal {V}}\,}}(S)(D)\\ D\in {{\,\mathrm{\mathbf {Dom}_R}\,}} \end{array}}\mathfrak {q}_x=\bigcap _{\begin{array}{c} \mathfrak {q}\in {{\,\mathrm{Spec}\,}}(R[M])\\ \mathfrak {q}\supseteq S \end{array}}\mathfrak {q}={{\,\mathrm{rad}\,}}(S) \text{ and } {{\,\mathrm{\mathcal {V}}\,}}(S)={{\,\mathrm{\mathcal {V}}\,}}({{\,\mathrm{rad}\,}}(S)). \end{aligned}$$

Hence ${{\,\mathrm{\mathcal {V}}\,}}(S)$ is uniquely determined by its associated subset of ${{\,\mathrm{{\mathbb A}}\,}}^n_R$. $\square$

While we have defined closed subsets of ${{\,\mathrm{{\mathbb A}}\,}}_M$ by looking at all *R*-domains *D*, it actually suffices to look at algebraic closures $\overline{K_\mathfrak {p}}$ where $\mathfrak {p}\in {{\,\mathrm{Spec}\,}}(R)$. For $\mathfrak {p}\in {{\,\mathrm{Spec}\,}}(R)$, we write $K_\mathfrak {p}:={{\,\mathrm{Frac}\,}}(R/\mathfrak {p})$ for the fraction field of $R/\mathfrak {p}$.

#### Proposition 35

Let *X* be a subset of ${{\,\mathrm{{\mathbb A}}\,}}_M$. Then

$$\begin{aligned} {{\,\mathrm{\mathcal {I}}\,}}_X=\bigcap _{\mathfrak {p}\in {{\,\mathrm{Spec}\,}}(R)}\left\{ f\in R[M]\,\bigg |\, f_{\overline{K_{\mathfrak {p}}}}\in {{\,\mathrm{\mathcal {I}}\,}}_{X(\overline{K_{\mathfrak {p}}})}\right\} . \end{aligned}$$

#### Proof

Clearly, the inclusion $\subseteq$ holds. Let $f\in R[M]$ be such that $f_{\overline{K_{\mathfrak {p}}}}\in {{\,\mathrm{\mathcal {I}}\,}}_{X(\overline{K_{\mathfrak {p}}})}$ for all $\mathfrak {p}\in {{\,\mathrm{Spec}\,}}(R)$. Let *D* be an *R*-domain and let $\mathfrak {p}$ be the kernel of the homomorphism $R \rightarrow D$. Then there exists a field *L* containing ${{\,\mathrm{Frac}\,}}(D)$ and $\overline{K_{\mathfrak {p}}}$. By the Nullstellensatz, the fact that $f_{\overline{K_{\mathfrak {p}}}}\in {{\,\mathrm{\mathcal {I}}\,}}_{X(\overline{K_{\mathfrak {p}}})}$ implies that $f_L\in {{\,\mathrm{\mathcal {I}}\,}}_{X(L)}$. It follows that $f_D$ vanishes on *X*(*D*). $\square$

#### Corollary 36

A closed subset *X* of ${{\,\mathrm{{\mathbb A}}\,}}_M$ is uniquely determined by its values $X(\overline{K_\mathfrak {p}})$ where $\mathfrak {p}$ runs over ${{\,\mathrm{Spec}\,}}(R)$.

#### Proof

This follows from the previous proposition since $X={{\,\mathrm{\mathcal {V}}\,}}({{\,\mathrm{\mathcal {I}}\,}}_X)$. $\square$

The proof of Theorem 2 in Sect. 6 follows a divide-and-conquer strategy in which the following two lemmas and their generalisations to closed subsets of polynomial functors (Lemmas 64 and 65), play a crucial role.

#### Lemma 37

Let *R* be a ring with Noetherian spectrum and *r* an element of *R*. Let $\mathfrak {p}_1,\ldots ,\mathfrak {p}_k$ be the minimal primes of *R*/(*r*). Then two closed subsets $X,Y\subseteq {{\,\mathrm{{\mathbb A}}\,}}_M$ are equal if and only if $X_{R[1/r]}=Y_{R[1/r]}$ and $X_{R/\mathfrak {p}_i}=Y_{R/\mathfrak {p}_i}$ for all $i=1,\ldots ,k$.

#### Proof

Suppose that $X_{R[1/r]}=Y_{R[1/r]}$ and $X_{R/\mathfrak {p}_i}=Y_{R/\mathfrak {p}_i}$ for all $i=1,\ldots ,k$. Let *K* be an *R*-field and let $R\rightarrow K$ be the corresponding homomorphism. If the image of *r* in *K* is zero, then $R \rightarrow K$ factors via $R/\mathfrak {p}_i$ for some $i =1, \ldots , k$ and hence *K* is a $(R/\mathfrak {p}_i)$-domain. In this case, we have $X(K) = X_{R/\mathfrak {p}_i}(K)= Y_{R/\mathfrak {p}_i}(K)=Y(K)$. If the image of *r* in *K* is nonzero, then *K* naturally is an *R*[1/*r*]-field. In this case, we have $X(K) = X_{R[1/r]}(K)= Y_{R[1/r]}(K)=Y(K)$. So $X=Y$ by Corollary 36. $\square$

#### Lemma 38

Let $R\subseteq R'$ be a finite extension of domains and let $X,Y\subseteq {{\,\mathrm{{\mathbb A}}\,}}_M$ be closed subsets. Then $X=Y$ if and only if $X_{R'}=Y_{R'}$.

#### Proof

The extension $R\subseteq R'$ satisfies lying over, i.e., for every prime $\mathfrak {p}\in {{\,\mathrm{Spec}\,}}(R)$ there is a prime $\mathfrak {q}\in {{\,\mathrm{Spec}\,}}(R')$ with $\mathfrak {p}=\mathfrak {q}\cap R$. The lemma follows by Corollary 36. $\square$

### Noetherianity of ${{\,\mathrm{{\mathbb A}}\,}}_M$

We now prove Proposition 1. Thus let *R* be a ring.

#### Lemma 39

If ${{\,\mathrm{Spec}\,}}(R)$ is Noetherian, then so is ${{\,\mathrm{Spec}\,}}(R[x])$.

#### Proof

This is an application of [12, Theorem 1.1] with trivial group. $\square$

#### Lemma 40

Assume that ${{\,\mathrm{Spec}\,}}(R)$ is Noetherian and set $N:=R^n$. Then ${{\,\mathrm{{\mathbb A}}\,}}_{N}$ is Noetherian, i.e., any chain $X_1 \supseteq X_2 \supseteq \cdots$ of closed subsets of ${{\,\mathrm{{\mathbb A}}\,}}_{N}$ stabilises eventually.

#### Proof

Consider the chain ${{\,\mathrm{\mathcal {I}}\,}}_{X_1}\subseteq {{\,\mathrm{\mathcal {I}}\,}}_{X_2}\subseteq \cdots$ of radical ideals in $R[N] \cong R[x_1,\ldots ,x_n]$. Since the latter ring has a topological spectrum, this chain stabilises. Since $X_i={{\,\mathrm{\mathcal {V}}\,}}({{\,\mathrm{\mathcal {I}}\,}}_{X_i})$, so does the chain $X_1 \subseteq X_2 \subseteq \cdots$. $\square$

#### Proof of Proposition 1

Let *R* be a ring with Noetherian spectrum, let *M* be a finitely generated *R*-module, and let $X_1 \supseteq X_2 \supseteq \cdots$ be a chain of closed subsets of ${{\,\mathrm{{\mathbb A}}\,}}_M$. Since *M* is finitely generated, there exists a surjective *R*-module homomorphism $\varphi :N:=R^n \rightarrow M$ for some *n*. This defines a (linear) polynomial law $N \rightarrow M$ and so a continuous map ${{\,\mathrm{{\mathbb A}}\,}}_N \rightarrow {{\,\mathrm{{\mathbb A}}\,}}_M$. Set $Y_i:= \varphi ^{-1}(X_i)$, which is the closed subset of ${{\,\mathrm{{\mathbb A}}\,}}_N$ such that $Y_i(D) = (1 \otimes \varphi )^{-1} (X_i(D))$ for all *R*-domains *D*. By Lemma 40, the chain $Y_1 \supseteq Y_2 \supseteq \cdots$ stabilises, i.e., $Y_n=Y_{n+1}$ for all $n \gg 0$. So, since $1 \otimes \varphi :D \otimes N \rightarrow D \otimes M$ is surjective for every *R*-domain *D*, we have $X_i(D)=(1 \otimes \varphi )(Y_i(D))$ for every *i* and *D*, and therefore $X_n=X_{n+1}$ for all $n\gg 0$. $\square$

#### Remark 41

If two ideals *I* and *J* in *R*[*M*] define the same closed subset in ${{\,\mathrm{Spec}\,}}(R[M])$, then they have the same radical and hence define the same closed subset in ${{\,\mathrm{{\mathbb A}}\,}}_M$. But it could possibly happen that two ideals that define the same closed subset in ${{\,\mathrm{{\mathbb A}}\,}}_M$ do *not* define the same closed subset in ${{\,\mathrm{Spec}\,}}(R[M])$. In particular, the proof above does not show that ${{\,\mathrm{Spec}\,}}(R[M])$ is a Noetherian topological space. Indeed, we don’t know whether this is the case.

#### Question 42

Suppose that ${{\,\mathrm{Spec}\,}}(R)$ is Noetherian and let *M* be a finitely generated *R*-module. Is ${{\,\mathrm{Spec}\,}}(R[M])$ Noetherian? Is the map from radical ideals of *R*[*M*] to closed subsets of ${{\,\mathrm{{\mathbb A}}\,}}_M$ a bijection?

### Dimension

#### Proposition 43

Let *R* be a domain, let *M* be a finitely generated *R*-module and let *X* be a closed subset of ${{\,\mathrm{{\mathbb A}}\,}}_M$. Then the function

$$\begin{aligned} {{\,\mathrm{Spec}\,}}(R)&\rightarrow {{\,\mathrm{{\mathbb Z}}\,}}_{\ge -1}\\ \mathfrak {p}&\mapsto \dim _{\overline{K_\mathfrak {p}}}( X(\overline{K_\mathfrak {p}})) \end{aligned}$$

is constant in some open dense subset ${{\,\mathrm{Spec}\,}}(R[1/r])$ of ${{\,\mathrm{Spec}\,}}(R)$.

#### Proof

By Lemma 8, there exists a nonzero $r\in R$ such that $R[1/r] \otimes M$ is free. It suffices to prove the statement for the domain *R*[1/*r*], the *R*[1/*r*]-module $R[1/r] \otimes M$ and the closed subset $X_{R[1/r]}$ of ${{\,\mathrm{{\mathbb A}}\,}}_{R[1/r]\otimes M}$. So we may assume that *M* is free, say of rank *m*, and so *X* is a closed subset of ${{\,\mathrm{{\mathbb A}}\,}}^m_R$; let $I \subseteq R[x_1,\ldots ,x_m]$ be its vanishing ideal. Choose an arbitrary monomial order on monomials in $x_1,\ldots ,x_m$. For each nonzero $r \in R$, let $M_r$ be the set of leading monomials of *monic* polynomials in $R[1/r] \otimes I$; this is an upper ideal in the monoid of monomials. By Dickson’s lemma, there exists an *r* such that $M_r$ is inclusion-wise maximal. Choose monic polynomials $f_1,\ldots ,f_k \in R[1/r][x_1,\ldots ,x_n]$ whose leading monomials generate the upper ideal $M_r$. Then $f_1,\ldots ,f_k$ generate the ideal $R[1/r] \otimes I$—indeed, otherwise there would be some element *f* in the latter ideal whose leading monomial is not divisible by any of the leading monomials of the $f_i$; and letting $r'$ be the leading coefficient of *f* we would find that $M_{rr'}$ strictly contains $M_r$, a contradiction. Moreover, again by maximality of $M_r$, the $f_i$ satisfy Buchberger’s criterion: every S-polynomial of them reduces to zero modulo $f_1,\ldots ,f_k$ when working over $R[1/r][x_1,\ldots ,x_m]$. Then for each $\mathfrak {p}\in {{\,\mathrm{Spec}\,}}(R[1/r])$, the images of the $f_i$ generate the ideal $K_\mathfrak {p}\otimes I=K_\mathfrak {p}\otimes _{R[1/r]} (R[1/r] \otimes I)$; and still satisfy Buchberger’s criterion. Hence these images form a Gröbner basis, and since the dimension of $X(\overline{K_\mathfrak {p}})$ can be read of from the set of leading monomials, that dimension is constant for $\mathfrak {p}\in {{\,\mathrm{Spec}\,}}(R[1/r])$. $\square$

#### Proposition 44

Let *R* be a domain, *M* a finitely generated *R*-module, and *X* a closed subset of ${{\,\mathrm{{\mathbb A}}\,}}_M$. Then there exists a nonzero $r \in R$ such that the following holds: for any $f \in R[M]$, if *f* vanishes identically on $X(\overline{K})$, then *f* vanishes identically on $X(\overline{K_\mathfrak {p}})$ for all $\mathfrak {p}\in {{\,\mathrm{Spec}\,}}(R[1/r])$.

#### Proof

As in the previous proof, it suffices to prove the statement in the case that *M* is free of rank *m*. Let $I \subseteq R[x_1,\ldots ,x_m]$ be the vanishing ideal of *X*. This time, for each nonzero $r\in R$, let $M_r$ be the set of leading monomials of *monic* polynomials in $R[1/r][x_1,\ldots ,x_m]$ *some power of which* lies in $R[1/r] \otimes I$. Choose *r* such that $M_r$ is maximal, and $f_1,\ldots ,f_k \in R[1/r][x_1,\ldots ,x_m]$ monic, whose powers lie in $R[1/r] \otimes I$, and whose leading monomials generate the upper ideal $M_r$. Then the images of $f_1,\ldots ,f_k$ form a Gröbner basis of the radical ideal of $K \otimes I$. Now assume that $f \in R[M]$ vanishes identically on $X(\overline{K})$, and let *g* be the image of *f* in $R[1/r][x_1,\ldots ,x_m]$. Then by the Nullstellensatz, some power of *g* reduces to zero modulo $f_1,\ldots ,f_k$. But then that reduction holds modulo $\mathfrak {p}$ for every $\mathfrak {p}\in {{\,\mathrm{Spec}\,}}(R[1/r])$, so *g* vanishes identically on $X(\overline{K_{\mathfrak {p}}})$ for all such $\mathfrak {p}$. $\square$

## Polynomial functors and their properties

### Polynomial functors over a ring

For reasons that will become clear later, we will only be interested in polynomial functors from the category ${{\,\mathrm{\mathbf {fgfMod}_R}\,}}$ of finitely generated free *R*-modules into either ${{\,\mathrm{\mathbf {Mod}_R}\,}}$ or ${{\,\mathrm{\mathbf {fgMod}_R}\,}}$.

#### Definition 45

A polynomial functor $P:{{\,\mathrm{\mathbf {fgfMod}_R}\,}}\rightarrow {{\,\mathrm{\mathbf {Mod}_R}\,}}$ consists of an object $P(U) \in {{\,\mathrm{\mathbf {Mod}_R}\,}}$ for each object $U\in {{\,\mathrm{\mathbf {fgfMod}_R}\,}}$ and a polynomial law

$$\begin{aligned} P_{U,V}:{{\,\mathrm{Hom}\,}}(U,V) \rightarrow {{\,\mathrm{Hom}\,}}(P(U),P(V)) \end{aligned}$$

for each $U,V \in {{\,\mathrm{\mathbf {fgfMod}_R}\,}}$ such that the diagram 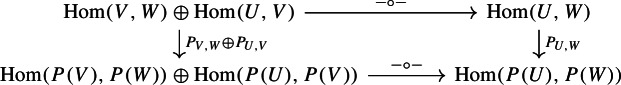 commutes for every $U,V,W \in {{\,\mathrm{\mathbf {fgfMod}_R}\,}}$. Here the bilinear horizontal polynomial laws are given as in Remark 15. Moreover, for every $U \in {{\,\mathrm{\mathbf {fgfMod}_R}\,}}$, we require that $P_{U,U}({{\,\mathrm{id}\,}}_U) = {{\,\mathrm{id}\,}}_{P(U)}$ and we require that *P* has *finite degree*, i.e., there is a uniform bound $d \in {{\,\mathrm{{\mathbb Z}}\,}}_{\ge 0}$ such that for all *U*, *V* the polynomial law $P_{U,V}$ has degree at most *d*.

Polynomial functors ${{\,\mathrm{\mathbf {fgfMod}_R}\,}}\rightarrow {{\,\mathrm{\mathbf {Mod}_R}\,}}$ form an Abelian category ${{\,\mathrm{\mathbf {PF}_R}\,}}$ in which a morphism $\alpha :Q \rightarrow P$ is given by an *R*-linear map $\alpha _U:Q(U) \rightarrow P(U)$ for each $U \in {{\,\mathrm{\mathbf {fgfMod}_R}\,}}$ such that the diagram of polynomial laws 
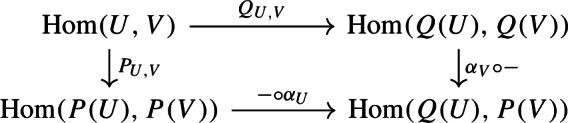
 commutes for all *U*, *V*. Note that post-composing with $\alpha _V$ and pre-composing with $\alpha _U$ are *R*-linear maps and hence, indeed, (linear) polynomial laws.

For every *R*-algebra *A* and *R*-modules *U*, *V*, *W*, let $-\circ _A-$ be the *A*-bilinear extension of the *R*-bilinear composition maps $-\circ -:{{\,\mathrm{Hom}\,}}(V,W)\times {{\,\mathrm{Hom}\,}}(U,V)\rightarrow {{\,\mathrm{Hom}\,}}(U,W)$. So $(-\circ _A-)_A$ is the polynomial law extending $-\circ -$. Then the diagram above says that

1 $$\begin{aligned} P_{U,V,A}(\varphi ) \circ _A(1\otimes \alpha _U) =(1 \otimes \alpha _V )\circ _A Q_{U,V,A}(\varphi ) \end{aligned}$$

for all *R*-algebras *A* and $\varphi \in A\otimes {{\,\mathrm{Hom}\,}}(U,V)$. Note that to check that the diagram commutes, it suffices to check that this equality holds for $A=R[x_1,\ldots ,x_n]$ and $\varphi =x_1\otimes \varphi _1+\cdots +x_n\otimes \varphi _n$ where $\varphi _1,\ldots ,\varphi _n$ is a basis of ${{\,\mathrm{Hom}\,}}(U,V)$.

Recall that for all *R*-modules *U*, *V*, there is a natural *A*-linear map

$$\begin{aligned} A \otimes {{\,\mathrm{Hom}\,}}(U,V) \rightarrow {{\,\mathrm{Hom}\,}}_A(A \otimes U,A \otimes V). \end{aligned}$$

For $U,V \in {{\,\mathrm{\mathbf {fgfMod}_R}\,}}$, this map is an isomorphism. Thus an element $\varphi$ of $A \otimes {{\,\mathrm{Hom}\,}}(U,V)$ can be thought of as an “element of ${{\,\mathrm{Hom}\,}}(U,V)$ with coordinates in *A*”. Viewing $Q_{U,V,A}(\varphi ),P_{U,V,A}(\varphi )$ as maps, (1) implies that the diagram 
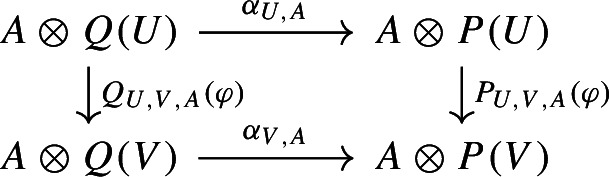
 commutes; here $\alpha _{U,A}$ is the *A*-linear extension of $\alpha _U$. When *A* is a polynomial ring over *R*, the map

$$\begin{aligned} A \otimes {{\,\mathrm{Hom}\,}}(Q(U),P(V)) \rightarrow {{\,\mathrm{Hom}\,}}_A(A \otimes Q(U),A \otimes P(V)) \end{aligned}$$

is injective and so the reverse implication also holds. So the family $(\alpha _U)_U$ is a morphism of polynomial functors if and only if the last diagram above commutes for all $A,U,V\varphi$. This is closer to the definition of polynomial functors over infinite fields, and generalises as follows.

#### Definition 46

Let *P*, *Q* be polynomial functors. We define a *polynomial transformation* $\alpha :Q\rightarrow P$ be a rule assigning to every $U\in {{\,\mathrm{\mathbf {fgfMod}_R}\,}}$ a polynomial law $\alpha _U:Q(U) \rightarrow P(U)$ such that the last diagram above commutes for all *R*-algebras *A* and $\varphi \in A\otimes {{\,\mathrm{Hom}\,}}(U,V)$.

Just like polynomial laws generalise *R*-module homomorphisms, and the latter are precisely the linear polynomial laws, polynomial transformations generalise morphisms of polynomial laws, and the latter are precisely the linear polynomial transformations.

#### Remark 47

If *R* is an infinite field, then a polynomial functor

$$\begin{aligned} P:{{\,\mathrm{\mathbf {fgfMod}_R}\,}}\rightarrow {{\,\mathrm{\mathbf {fgMod}_R}\,}}={{\,\mathrm{\mathbf {fgfMod}_R}\,}}\end{aligned}$$

is a the same thing as a functor from the category of finite-dimensional *R*-vector spaces to itself such that for all $U,V \in {{\,\mathrm{\mathbf {fgfMod}_R}\,}}$ the map

$$\begin{aligned} P_{U,V}:{{\,\mathrm{Hom}\,}}(U,V) \rightarrow {{\,\mathrm{Hom}\,}}(P(U),P(V)) \end{aligned}$$

is a polynomial map. This is the set-up in [8]. If *R* is a field but not necessarily infinite, then a polynomial functor ${{\,\mathrm{\mathbf {fgfMod}_R}\,}}\rightarrow {{\,\mathrm{\mathbf {fgfMod}_R}\,}}$ is a strict polynomial functor in the sense of Friedlander–Suslin [15].

Many of our proofs will involve passing to the case of (infinite) fields and invoking arguments from [8]. This is facilitated by the following construction.

#### Definition 48

(*Base change*). Let *B* be an *R*-algebra and let $P:{{\,\mathrm{\mathbf {fgfMod}_R}\,}}\rightarrow {{\,\mathrm{\mathbf {Mod}_R}\,}}$ be a polynomial functor. Then *P* induces a polynomial functor $P_B$ from ${{\,\mathrm{\mathbf {fgfMod}_B}\,}}$ to ${{\,\mathrm{\mathbf {Mod}_B}\,}}$ as follows: first, for each finitely generated free *B*-module *U* fix a *B*-module isomorphism $\psi _U:U \rightarrow B \otimes U_R$, where $U_R$ is a free *R*-module of the same *R*-rank as the *B*-rank of *U*. Then, set $P_B(U):=B \otimes P(U_R)$. Next, for each *B*-algebra *A*, we need to assign to every $\varphi \in A \otimes _B {{\,\mathrm{Hom}\,}}_B(U,V)$ an image in $A \otimes {{\,\mathrm{Hom}\,}}_B(P_B(U),P_B(V))$. For this, note that

$$\begin{aligned} \begin{array}{rll} A \otimes _B {{\,\mathrm{Hom}\,}}_B(U,V) &{}\cong A \otimes _B {{\,\mathrm{Hom}\,}}_B(B \otimes U_R,B \otimes V_R) &{}\\ &{}\cong A \otimes _B (B \otimes {{\,\mathrm{Hom}\,}}(U_R,V_R))\\ &{}\cong A \otimes {{\,\mathrm{Hom}\,}}(U_R,V_R), \end{array} \end{aligned}$$

where the isomorphism in the first step is $1_A \otimes _B (\psi _V \circ -\circ \psi _U^{-1})$ and the second isomorphism follows from the freeness of $U_R$ and $V_R$. Via these isomorphisms, $\varphi$ is mapped to an element of $A \otimes {{\,\mathrm{Hom}\,}}(U_R,V_R)$. Applying $P_{U_R,V_R,A}$ to this element yields an element of $A \otimes {{\,\mathrm{Hom}\,}}(P(U_R),P(V_R)) \cong A \otimes _B (B \otimes {{\,\mathrm{Hom}\,}}(P(U_R),P(V_R)))$, and applying the natural map $B \otimes {{\,\mathrm{Hom}\,}}(P(U_R),P(V_R)) \rightarrow {{\,\mathrm{Hom}\,}}_B(B \otimes P(U_R),B \otimes P(V_R))$ in the second factor (which may not be an isomorphism since $P(U_R),P(V_R)$ need not be free) yields an element of $A \otimes _B {{\,\mathrm{Hom}\,}}_B(P_B(U),P_B(V))$. It is straightforward to check that $P_B$ thus defined is a polynomial functor from ${{\,\mathrm{\mathbf {fgfMod}_B}\,}}$ to ${{\,\mathrm{\mathbf {Mod}_B}\,}}$. A different choice of isomorphisms $\psi _U$ yields a different but isomorphic polynomial functor $P_B$.

#### Remark 49

In this construction we have made use of the fact that *P* is a polynomial functor from finitely generated *free* *R*-modules to *R*-modules. The choice of $\psi _U$’s could have been avoided as follows: instead of working with ${{\,\mathrm{\mathbf {fgfMod}_R}\,}}$, we could have worked with the category whose objects are finite sets and whose morphisms $J \rightarrow I$ are given by $I \times J$ matrices with entries in *R*. Then $P_{J,I}$ would have been a polynomial law from the module of $I \times J$ matrices to ${{\,\mathrm{Hom}\,}}(P(J),P(I))$. However, the set-up we chose stresses better that we are interested in phenomena that do *not* depend on a choice of basis in our free modules.

#### Definition 50

A polynomial functor $P:{{\,\mathrm{\mathbf {fgfMod}_R}\,}}\rightarrow {{\,\mathrm{\mathbf {Mod}_R}\,}}$ is called homogeneous of degree *d* if the polynomial law $P_{U,V}$ is homogeneous of degree *d* for each $U,V\in {{\,\mathrm{\mathbf {fgfMod}_R}\,}}$.

Every polynomial functor $P:{{\,\mathrm{\mathbf {fgfMod}_R}\,}}\rightarrow {{\,\mathrm{\mathbf {Mod}_R}\,}}$ is a direct sum $P_0 \oplus \cdots \oplus P_d$, where $P_i:{{\,\mathrm{\mathbf {fgfMod}_R}\,}}\rightarrow {{\,\mathrm{\mathbf {Mod}_R}\,}}$ is the homogeneous polynomial functor of degree *i* given on objects by $P_i(V)=\{v\in P(V)\mid P_{V,V,R[t]}(t\otimes {{\,\mathrm{id}\,}}_V)(v)=t^i\otimes v\}$; and $P_{i,U,V}$ is the restriction of the degree-*i* component of the polynomial law $P_{U,V}$ to $P_i(U)$. Here we identify $R[t]\otimes {{\,\mathrm{Hom}\,}}(P(V),P(V))$ with ${{\,\mathrm{Hom}\,}}(P(V),R[t]\otimes P(V))$.

### Duality

#### Definition 51

Let $P:{{\,\mathrm{\mathbf {fgfMod}_R}\,}}\rightarrow {{\,\mathrm{\mathbf {Mod}_R}\,}}$ be a polynomial functor over *R*. Then we obtain another polynomial functor $P^*:{{\,\mathrm{\mathbf {fgfMod}_R}\,}}\rightarrow {{\,\mathrm{\mathbf {Mod}_R}\,}}$ by setting, for each $V \in {{\,\mathrm{\mathbf {fgfMod}_R}\,}}$, $P^*(V):=P(V^*)^*={{\,\mathrm{Hom}\,}}(P(V^*),R)$ and for each $\varphi \in A \otimes {{\,\mathrm{Hom}\,}}(U,V)$,

$$\begin{aligned} P^*_{U,V,A}(\varphi ):=P_{V^*,U^*,A}(\varphi ^*)^*, \end{aligned}$$

where $\varphi ^*$ is the image of $\varphi$ under the natural isomorphism

$$\begin{aligned} A \otimes {{\,\mathrm{Hom}\,}}(U,V) \cong A \otimes {{\,\mathrm{Hom}\,}}(V^*,U^*) \end{aligned}$$

(here we use that *U*, *V* are free) and the outermost $*$ again represents a dual.

The dual functor $P^*$ of *P* has the same degree as *P* and will play a role in Sect. 6.10. To avoid having too many stars, we will there think of it as the functor that sends $V^*$ to $P(V)^*$. If *P* takes values in ${{\,\mathrm{\mathbf {fgfMod}_R}\,}}$, then $(P^*)^*$ is canonically isomorphic to *P*.

### Shifting

Let *U* be a finitely generated free *R*-module.

#### Definition 52

We define the shift functor ${{\,\mathrm{Sh}\,}}_U:{{\,\mathrm{\mathbf {fgfMod}_R}\,}}\rightarrow {{\,\mathrm{\mathbf {fgfMod}_R}\,}}$ that sends $V\mapsto U \oplus V$ and $\varphi \mapsto {{\,\mathrm{id}\,}}_U\oplus \,\varphi$. For a polynomial functor $P:{{\,\mathrm{\mathbf {fgfMod}_R}\,}}\rightarrow {{\,\mathrm{\mathbf {fgMod}_R}\,}}$ we set ${{\,\mathrm{Sh}\,}}_U(P):=P \circ {{\,\mathrm{Sh}\,}}_U$, called the *shift of P by U*.

#### Lemma 53

The composition ${{\,\mathrm{Sh}\,}}_U(P)$ is again a polynomial functor ${{\,\mathrm{\mathbf {fgfMod}_R}\,}}\rightarrow {{\,\mathrm{\mathbf {fgMod}_R}\,}}$, the projection $U \oplus V \rightarrow V$ yields a surjection of polynomial functors ${{\,\mathrm{Sh}\,}}_U(P) \rightarrow P$ and inclusion the $V \rightarrow U \oplus V$ yields a section $P \rightarrow {{\,\mathrm{Sh}\,}}_U(P)$ to that surjection. In particular, ${{\,\mathrm{Sh}\,}}_U(P) \cong P \oplus ({{\,\mathrm{Sh}\,}}_U(P)/P)$. Furthermore, ${{\,\mathrm{Sh}\,}}_U(P)/P$ has degree strictly smaller than the degree of *P*.

#### Proof

The proof in [8, Lemma 14] (in the case where *R* is an infinite field) carries over to the current more general setting. $\square$

### Dimension functions of polynomial functors

Let $P:{{\,\mathrm{\mathbf {fgfMod}_R}\,}}\rightarrow {{\,\mathrm{\mathbf {fgMod}_R}\,}}$ be a polynomial functor. For $\mathfrak {p}\in {{\,\mathrm{Spec}\,}}(R)$, set $f_\mathfrak {p}(n):=\dim _{K_{\mathfrak {p}}}(K_\mathfrak {p}\otimes P(R^n))$. It turns out that these functions are polynomials in *n*, and depend semicontinuously on $\mathfrak {p}$. To formalise this semicontinuity, we order polynomials in ${{\,\mathrm{{\mathbb Z}}\,}}[x]$ by $f \ge g$ if $f(n) \ge g(n)$ for all $n \gg 0$; this is the lexicographic order on coefficients.

#### Proposition 54

For each $\mathfrak {p}\in {{\,\mathrm{Spec}\,}}(R)$ the function $f_\mathfrak {p}:{{\,\mathrm{{\mathbb Z}}\,}}_{\ge 0} \rightarrow {{\,\mathrm{{\mathbb Z}}\,}}_{\ge 0}$ is a polynomial with integral coefficients of degree at most the degree of *P*. Furthermore, the map $\mathfrak {p}\mapsto f_\mathfrak {p}$ is upper semicontinuous on ${{\,\mathrm{Spec}\,}}(R)$ in a strong sense: both the sets $\{\mathfrak {p}\mid f_\mathfrak {p}\ge f \}$ and $\{\mathfrak {p}\mid f_\mathfrak {p}> f\}$ are closed for all $f \in {{\,\mathrm{{\mathbb Z}}\,}}[x]$.

#### Proof

We proceed by induction on the degree of *P*. If *P* has degree 0, then $P(R^n)$ is a fixed *R*-module *U*, and $f_\mathfrak {p}$ is the constant polynomial that maps *n* to $\dim _{K_\mathfrak {p}}(K_\mathfrak {p}\otimes U)$. In this case, if $f \in {{\,\mathrm{{\mathbb Z}}\,}}[x]$ has positive degree, then $f_\mathfrak {p}> f$ and $f_\mathfrak {p}\ge f$ are either both trivially true for all $\mathfrak {p}$ or both trivially false for $\mathfrak {p}$ (depending on the sign of the leading coefficient of *f*), so we need only look at constant *f*.

In this case, the result is classical; we recall the argument. Let $R^n \rightarrow U$ be a surjective *R*-module homomorphism, and let *N* be its kernel. Since tensoring with $K_\mathfrak {p}$ is right-exact, $1 \otimes N$ spans the kernel of the surjection $K_\mathfrak {p}^n \rightarrow K_\mathfrak {p}\otimes U$ for each $\mathfrak {p}$.

The statement that $\dim _{K_\mathfrak {p}}(K_\mathfrak {p}\otimes U)$ is upper semicontinuous is therefore equivalent to the statement that dimension of the span of $1 \otimes N$ in $K_\mathfrak {p}^n$ is lower semicontinuous. And indeed, the locus where this dimension is less than *k* is defined by the vanishing of all $k \times k$ subdeterminants of all $k \times n$ matrices (with entries in *R*) whose rows are *k* elements of *N*.

For the induction step, assume that the proposition is true for all polynomial functors of degree $<d$ and assume that *P* has degree $d \ge 1$. Then consider the functor ${{\,\mathrm{Sh}\,}}_R(P)$, which by Lemma 53 is isomorphic to $P \oplus Q$ for $Q:={{\,\mathrm{Sh}\,}}_R(P)/P$ of degree $<d$.

By the induction hypothesis, the proposition holds for *Q*: the function $g_\mathfrak {p}(n):= \dim _{K_\mathfrak {p}}(K_\mathfrak {p}\otimes Q(R^n))$ equals a polynomial with integral coefficients for all $n \ge 0$, and $\mathfrak {p}\mapsto g_\mathfrak {p}$ is semicontinuous. Now we have

$$\begin{aligned} \begin{array}{rll} f_\mathfrak {p}(n+1)&{}= \dim _{K_\mathfrak {p}}(K_\mathfrak {p}\otimes P(R^1 \oplus R^n))&{}\\ &{}=\dim _{K_\mathfrak {p}} (K_\mathfrak {p}\otimes P(R^n)) + \dim _{K_\mathfrak {p}} (K_\mathfrak {p}\otimes Q(R^n))&{}=f_\mathfrak {p}(n) + g_\mathfrak {p}(n). \end{array} \end{aligned}$$

This means that $f_\mathfrak {p}(n)$ is the unique polynomial with $(\Delta f_\mathfrak {p})(n):=f_\mathfrak {p}(n+1)-f_\mathfrak {p}(n)=g_\mathfrak {p}(n)$ for $n \ge 0$ and $f_{\mathfrak {p}}(0)=\dim _{K_\mathfrak {p}}(K_\mathfrak {p}\otimes P(0))$; this $f_\mathfrak {p}$ has integral coefficients and degree at most *d*.

For the semi-continuity statement, note that $f_\mathfrak {p}\ge f$ is equivalent to either $g_\mathfrak {p}=\Delta f_\mathfrak {p}> \Delta f$, or else $g_\mathfrak {p}\ge \Delta f$ and moreover $f_\mathfrak {p}(0) \ge f(0)$. Both possibilities are closed conditions on $\mathfrak {p}$. Similarly, $f_\mathfrak {p}> f$ is equivalent to either $g_\mathfrak {p}> \Delta f$ or else $g_\mathfrak {p}\ge \Delta f$ and $f_{\mathfrak {p}}(0)> f(0)$, which, again, are closed conditions. $\square$

### Local freeness

We now generalise Lemma 8 to polynomial functors.

#### Proposition 55

Let *R* be a domain, $P:{{\,\mathrm{\mathbf {fgfMod}_R}\,}}\rightarrow {{\,\mathrm{\mathbf {fgMod}_R}\,}}$ a polynomial functor and *S* a subobject of *P* in the larger category of polynomial functors ${{\,\mathrm{\mathbf {fgfMod}_R}\,}}\rightarrow {{\,\mathrm{\mathbf {Mod}_R}\,}}$. Then there exists a nonzero $r \in R$ such that $R[1/r] \otimes S(U)$ and $R[1/r] \otimes P(U)$ are finitely generated free *R*[1/*r*]-modules for all $U \in {{\,\mathrm{\mathbf {fgfMod}_R}\,}}$, and the latter is a direct sum of the former and another free *R*[1/*r*]-module.

Note that we do not claim that the complement is itself the evaluation of another subobject; i.e., $S_{R[1/r]}$ needs not be a summand of $P_{R[1/r]}$ in the category of polynomial functors over *R*[1/*r*].

#### Proof

Again, we proceed by induction on the degree of *P*. If *P* has degree 0, then so does *S* and then the statement is just Lemma 8. Suppose that the degree of *P* is $d>0$ and that the proposition holds for all polynomial functors of degree less than *d*.

By Lemma 53, for each *n* we have

$$\begin{aligned} P(R^{n+1})=P(R^n) \oplus Q(R^n) \end{aligned}$$

where $Q={{\,\mathrm{Sh}\,}}_R(P)/ P$ has degree $<d$. Similarly, we have

$$\begin{aligned} S(R^{n+1})=S(R^n) \oplus N(R^n) \end{aligned}$$

where $N={{\,\mathrm{Sh}\,}}_R(S) / S \subseteq Q$. It follows that

$$\begin{aligned} P(R^n)&=P(0) \oplus Q(0) \oplus Q(R^1) \oplus \cdots \oplus Q(R^{n-1}) \text { and}\\ S(R^n)&=S(0) \oplus N(0)\oplus N(R^1) \oplus \cdots \oplus N(R^{n-1}). \end{aligned}$$

Now by Lemma 8 there exists a nonzero $r_0$ such that $R[1/r_0] \otimes P(0)$ is the direct sum of a free $R[1/r_0]$-module and $R[1/r_0] \otimes S(0)$, which is also free. And by the induction hypothesis there exists a nonzero $r_1 \in R$ such that for each *m*, $R[1/r_1] \otimes Q(R^m)$ is a direct sum of two free $R[1/r_1]$-modules, one of which is $R[1/r_1] \otimes N(R^m)$. Then $r:=r_0 \cdot r_1$ does the trick for the pair *P*, *S*. $\square$

### The Friedlander–Suslin lemma

The Friedlander–Suslin lemma relates polynomial functors of bounded degree to representations of certain associative algebras called *Schur Algebras*. To introduce these, let $U \in {{\,\mathrm{\mathbf {fgfMod}_R}\,}}$ and let $d\ge 1$ be an integer. The bilinear polynomial law

$$\begin{aligned} - \circ -:{{\,\mathrm{End}\,}}(U) \times {{\,\mathrm{End}\,}}(U) \rightarrow {{\,\mathrm{End}\,}}(U) \end{aligned}$$

given by composition yields an algebra homomorphism

$$\begin{aligned} R[{{\,\mathrm{End}\,}}(U)] \rightarrow R[{{\,\mathrm{End}\,}}(U) \times {{\,\mathrm{End}\,}}(U)] \cong R[{{\,\mathrm{End}\,}}(U)] \otimes R[{{\,\mathrm{End}\,}}(U)] \end{aligned}$$

which maps the part $R[{{\,\mathrm{End}\,}}(U)]_{\le d}$ of degree $\le d$ into

$$\begin{aligned} \sum _{\begin{array}{c} a,b \ge 0\\ a+b \le d \end{array}} R[{{\,\mathrm{End}\,}}(U)]_a \otimes R[{{\,\mathrm{End}\,}}(U)]_b \subseteq R[{{\,\mathrm{End}\,}}(U)]_{\le d} \otimes R[{{\,\mathrm{End}\,}}(U)]_{\le d}. \end{aligned}$$

Taking the dual *R*-modules, we obtain a map

$$\begin{aligned}&R[{{\,\mathrm{End}\,}}(U)]_{\le d}^* \otimes R[{{\,\mathrm{End}\,}}(U)]_{\le d}^* \rightarrow (R[{{\,\mathrm{End}\,}}(U)]_{\le d} \otimes R[{{\,\mathrm{End}\,}}(U)]_{\le d})^* \\&\quad \rightarrow R[{{\,\mathrm{End}\,}}(U)]_{\le d}^*. \end{aligned}$$

We set $S_{\le d}(U):=R[{{\,\mathrm{End}\,}}(U)]_{\le d}^*$. The first map is, in fact, an isomorphism due to the fact that $S_{\le d}(U)$ is finitely generated and free as an *R*-module. Indeed, if *U* is free with basis $u_1,\ldots ,u_n$, then ${{\,\mathrm{End}\,}}(U)$ is free with basis $(E_{ij})_{i,j=1}^n$, where $E_{ij}u_k =\delta _{jk} u_i$, and $R[{{\,\mathrm{End}\,}}(U)]_{\le d}$ is free with basis the monomials $x^{\alpha }$ of degree $\le d$ in the coordinates $x_{ij}$ dual to the $E_{ij}$, and hence $R[{{\,\mathrm{End}\,}}(U)]_{\le d}^*$ is free with the dual basis $(s_\alpha )_\alpha$, where $\alpha$ runs over all multi-indices in ${{\,\mathrm{{\mathbb Z}}\,}}_{\ge 0}^{n\times n}$ such that $|\alpha |:=\sum _{i,j} \alpha _{i,j} \le d$. We let $-*-:S_{\le d}(U)\times S_{\le d}(U) \rightarrow S_{\le d}(U)$ be the bilinear map associated to the map above.

#### Definition 56

The *R*-module $S_{\le d}(U)$ with the bilinear map $-*-$ is called the *Schur algebra of degree* $\le d$ *on* *U*, and (given a basis of *U*), the basis $(s_{\alpha })_\alpha$ is called its *distinguished basis*.

The Schur algebra is associative (but not commutative unless $n=1$); this follows from the associativity of composition in ${{\,\mathrm{End}\,}}(U)$. Explicitly, the coefficient of $s_{\gamma }$ in the product $s_\alpha * s_{\beta }$ is computed as follows: First, expand the composition $(\sum _{ij} x_{ij} E_{ij}) \circ (\sum _{kl} y_{kl} E_{kl})$, where the $x_{ij}$ and $y_{kl}$ are variables, as $\sum _{i,l} (\sum _j x_{ij} y_{jl}) E_{il}=:\sum _{il} z_{il} E_{il}$. Then expand $z^\gamma$ as a polynomial in the $x_{ij}$ and the $y_{kl}$, and take the coefficient of the monomial $x^\alpha y^\beta$.

The map ${{\,\mathrm{End}\,}}(U) \rightarrow S_{\le d}(U)$ that sends $\varphi$ to the *R*-linear evaluation map

$$\begin{aligned} R[{{\,\mathrm{End}\,}}(U)]_{\le d} \rightarrow R,\quad f \mapsto f_R(\varphi ) \end{aligned}$$

is an injective homomorphism of associative *R*-algebras, so $S_{\le d}(U)$-modules *M* are, in particular, representations of the *R*-algebra ${{\,\mathrm{End}\,}}(U)$. In fact, they are precisely the *polynomial* ${{\,\mathrm{End}\,}}(U)$-representations of degree $\le d$, i.e., those for which the map ${{\,\mathrm{End}\,}}(U) \rightarrow {{\,\mathrm{End}\,}}(M)$ is not just a homomorphism of (noncommutative) *R*-algebras but also a polynomial law making certain diagrams commute. Since we will not need this interpretation, we skip the details.

Now suppose that *P* is a polynomial functor ${{\,\mathrm{\mathbf {fgfMod}_R}\,}}\rightarrow {{\,\mathrm{\mathbf {Mod}_R}\,}}$ of degree $\le d$. Then *P*(*U*) naturally carries the structure of an $S_{\le d}(U)$-module as follows: the polynomial law

$$\begin{aligned} P_{U,U}:{{\,\mathrm{End}\,}}(U) \rightarrow {{\,\mathrm{End}\,}}(P(U)) \end{aligned}$$

has degree $\le d$ and therefore we have

$$\begin{aligned} P_{U,U,R[x_{11},x_{12},\ldots ,x_{nn}]} \left( \sum _{i,j=1}^nx_{ij}\otimes E_{ij}\right) = \sum _{|\alpha | \le d} x^\alpha \otimes \varphi _\alpha \end{aligned}$$

for certain endomorphisms $\varphi _\alpha \in {{\,\mathrm{End}\,}}(P(U))$. Now the basis element $s_\alpha$ of $S_{\le d}(U)$ acts on *P*(*U*) via $\varphi _\alpha$; it can be shown that this construction is independent of the choice of basis of *U*.

#### Theorem 57

(Friedlander–Suslin lemma, [27, Théorème 7.2] and [15, Theorem 3.2]). Let $U \in {{\,\mathrm{\mathbf {fgfMod}_R}\,}}$ have rank $\ge d$. Then the association $P \mapsto P(U)$ is an equivalence of Abelian categories from the full subcategory of ${{\,\mathrm{\mathbf {PF}_R}\,}}$ consisting of polynomial functors ${{\,\mathrm{\mathbf {fgfMod}_R}\,}}\rightarrow {{\,\mathrm{\mathbf {Mod}_R}\,}}$ of degree $\le d$ to the category of $S_{\le d}(U)$-modules.

To conclude this section, we observe that Schur algebras behave well under base change: if *A* is an *R*-algebra, then we have a commuting diagram (up to natural isomorphisms):where the lower horizontal map is evaluation at $A \otimes U$ and the *A*-algebra $A \otimes S_{\le d}(U)$ is canonically isomorphic to the Schur algebra $S_{\le d}(A \otimes U)$ on the free *A*-module $A \otimes U$.

### Irreducibility in an open subset of ${{\,\mathrm{Spec}\,}}(R)$

Let *R* be a domain and let $P:{{\,\mathrm{\mathbf {fgfMod}_R}\,}}\rightarrow {{\,\mathrm{\mathbf {fgMod}_R}\,}}$ be a polynomial functor. As before, for each prime $\mathfrak {p}\in {{\,\mathrm{Spec}\,}}(R)$ we set $K_\mathfrak {p}:={{\,\mathrm{Frac}\,}}(R/\mathfrak {p})$; in particular, $K:=K_{(0)}$ is the fraction field of *R*. Recall that the base change functor yields a polynomial functor $P_{K_\mathfrak {p}}$ over the field $K_\mathfrak {p}$ for each $\mathfrak {p}\in {{\,\mathrm{Spec}\,}}(R)$, and also a polynomial functor $P_{\overline{K_{\mathfrak {p}}}}$ over the algebraic closure $\overline{K_{\mathfrak {p}}}$ of $K_{\mathfrak {p}}$. The goal of this section is to transfer certain properties of $P_{K}$ to $P_{K_\mathfrak {p}}$ for $\mathfrak {p}$ in an open dense subset of ${{\,\mathrm{Spec}\,}}(R)$.

#### Proposition 58

Let $\overline{Q}$ be an irreducible subobject of $P_{K}$ in the Abelian category of polynomial functors over *K* and assume that $\overline{Q}_{\overline{K}}$ is still irreducible. Then there exists a subobject *Q* of *P* in the category of polynomial functors ${{\,\mathrm{\mathbf {fgfMod}_R}\,}}\rightarrow {{\,\mathrm{\mathbf {Mod}_R}\,}}$ such that $Q_K=\overline{Q}$ and $Q_{\overline{K_\mathfrak {p}}}$ is an irreducible subobject of $P_{\overline{K_\mathfrak {p}}}$ in the Abelian category of polynomial functors over $\overline{K_\mathfrak {p}}$ for all primes $\mathfrak {p}$ in a dense open subset ${{\,\mathrm{Spec}\,}}(R[1/r]) \subseteq {{\,\mathrm{Spec}\,}}(R)$.

#### Remark 59

Note that we don’t require that *Q* is a functor into ${{\,\mathrm{\mathbf {fgMod}_R}\,}}$; we may not be able to guarantee this if *R* is not a Noetherian ring.

In order to prove this proposition, we use the following lemma.

#### Lemma 60

Let *A* be a (not necessarily commutative) associative *R*-algebra and *N* an *A*-module that is, as an *R*-module, finitely generated and free. Suppose that $\overline{K} \otimes N$ is an irreducible $(\overline{K} \otimes A)$-module. Then there exists a dense open subset ${{\,\mathrm{Spec}\,}}(R[1/r]) \subseteq {{\,\mathrm{Spec}\,}}(R)$ such that $\overline{K_\mathfrak {p}} \otimes N$ is an irreducible $(\overline{K_\mathfrak {p}} \otimes A)$-module for all $\mathfrak {p}\in {{\,\mathrm{Spec}\,}}(R[1/r])$.

#### Proof

Let $v_1,\ldots ,v_n$ be an *R*-basis of *N*. For each $j \in [n]$ and each $a \in A$ let $c_{a,i,j} \in R$ be the *structure constants* determined by

$$\begin{aligned} a v_j = \sum _i c_{a,i,j} v_i. \end{aligned}$$

For each $k=1,\ldots ,n-1$, we will construct a constructible subset $Z_k$ of the Grassmannian ${{\,\mathrm{Gr}\,}}_{R}(k,n)$ over *R* whose set of $\overline{K_\mathfrak {p}}$-points, for $\mathfrak {p}\in {{\,\mathrm{Spec}\,}}(R)$, is the set of *k*-dimensional $(\overline{K_\mathfrak {p}} \otimes A)$-submodules of $\overline{K_{\mathfrak {p}}} \otimes N$. The construction is as follows: for each $J \subseteq [n]$ of size *k* consider the $k \times n$ matrix $X_J$ whose entries on the columns labelled by *J* are a $k \times k$ identity matrix over *R* and whose other entries are variables $x_{ij},i \in [k], j \in [n] \setminus J$. Recall that ${{\,\mathrm{Gr}\,}}_{R}(k,n)$ has an open cover of affine spaces ${{\,\mathrm{{\mathbb A}}\,}}_{R,J}^{k \times (n-k)}$ over *R* on which the coordinates are precisely these $x_{ij}$ with $j \not \in J$. For $j \in J$ we write $x_{ij} \in \{0,1\}$ for the corresponding entry of $X_J$. Note that, for each $m=1,\ldots ,k$ and each $a \in A$, we have

$$\begin{aligned}&(1 \otimes a)\left( \sum _{j=1}^n x_{mj} \otimes v_j\right) \\&\quad = \sum _{i=1}^n \sum _{j=1}^n c_{a,i,j} x_{mj} \otimes v_i\in R\left[ x_{ij}\,\bigg |\, i\in [k],j\in [n]\setminus J\right] \otimes N \end{aligned}$$

and we define the row vector of coefficients

$$\begin{aligned} y_{a,m}:=\left( \sum _{j=1}^n c_{a,i,j} x_{mj}\right) _{i=1}^n \end{aligned}$$

with entries in the coordinate ring $R[x_{ij}\mid i\in [k],j\in [n]\setminus J]$ of ${{\,\mathrm{{\mathbb A}}\,}}_{R,J}^{k \times (n-k)}$.

Let $C_J$ be the closed subset of ${{\,\mathrm{{\mathbb A}}\,}}_{R,J}^{k \times (n-k)}$ defined by the vanishing of all $(k+1) \times (k+1)$-subdeterminants of the matrices

$$\begin{aligned} \begin{bmatrix} y_{a,m} \\ X_J \end{bmatrix} \end{aligned}$$

for all choices of $a \in A$ and $m=1,\ldots ,k$. For each prime $\mathfrak {p}\in {{\,\mathrm{Spec}\,}}(R)$, the subset $C_J(\overline{K_\mathfrak {p}}) \subseteq {{\,\mathrm{Gr}\,}}_R(k,n)(\overline{K_\mathfrak {p}})$ parameterises the *k*-dimensional $(\overline{K_{\mathfrak {p}}} \otimes A)$-submodules of $\overline{K_{\mathfrak {p}}} \otimes N\cong \overline{K_{\mathfrak {p}}}^{[n]}$ that map surjectively to $\overline{K_\mathfrak {p}}^J$. In particular, by the assumption that $\overline{K} \otimes N$ is still irreducible, the image of $C_J$ in ${{\,\mathrm{Spec}\,}}(R)$ does not contain the prime 0, for any *k* and any *k*-set $J \subseteq [n]$. In other words, the morphism $C_J \rightarrow {{\,\mathrm{Spec}\,}}(R)$ is not dominant. Set $Z_k:=\bigcup _{J \subseteq [n], |J|=k} \overline{C_J}$, a finite union of locally closed subsets of the Grassmannian. Then $Z_k \rightarrow {{\,\mathrm{Spec}\,}}(R)$ is still not dominant, and neither is $\left( \bigcup _{k=1}^{n-1}Z_k\right) \rightarrow {{\,\mathrm{Spec}\,}}(R)$. Hence there exists a nonzero $r \in R$ that lies in the vanishing ideal of the image; the open dense subset ${{\,\mathrm{Spec}\,}}(R[1/r]) \subseteq {{\,\mathrm{Spec}\,}}R$ then has the desired property. $\square$

#### Proof of Proposition 58

By the Friedlander–Suslin Lemma (Theorem 57) and the fact that the Schur algebra behaves well under base change, it suffices to prove the corresponding statement for all $d \in {{\,\mathrm{{\mathbb Z}}\,}}_{\ge 0}$, $U:=R^d$, and all $S_{\le d}(U)$-modules that are finitely generated over *R* (which, of course, is equivalent to being finitely generated as an $S_{\le d}(U)$-module).

So let *M* be a finitely generated $S_{\le d}(U)$-module and let $\overline{N}$ be an irreducible $(K \otimes S_{\le d}(U))$-submodule of $K \otimes M$ that remains irreducible when tensoring with $\overline{K}$. Define

$$\begin{aligned} N:=\{v \in M \mid 1 \otimes v \in \overline{N} \}. \end{aligned}$$

A straightforward computation shows that *N* is a (not necessarily finitely generated) $S_{\le d}(U)$-submodule of *M*.

By Lemma 8 there exist a nonzero $r \in R$ and elements $v_1,\ldots ,v_n \in N$ such that $R[1/r] \otimes N$ is a free *R*[1/*r*]-module with basis $1 \otimes v_1,\ldots ,1 \otimes v_n$. Then Lemma 60 applied with *R* equal to *R*[1/*r*] and *A* equal to $R[1/r] \otimes S_{\le d}(U)$ shows that $\overline{K_{\mathfrak {p}}} \otimes N$ is an irreducible $(\overline{K_{\mathfrak {p}}} \otimes S_{\le d}(U))$-submodule of $\overline{K_{\mathfrak {p}}} \otimes M$ for all $\mathfrak {p}$ in some nonempty open subset ${{\,\mathrm{Spec}\,}}R[1/(rs)] \subseteq {{\,\mathrm{Spec}\,}}(R[1/r]) \subseteq {{\,\mathrm{Spec}\,}}(R)$. $\square$

### Closed subsets of polynomial functors

Closed subsets of a polynomial functors play the role of affine varieties in finite-dimensional algebraic geometry. In this subsection, *P* is a fixed polynomial functor ${{\,\mathrm{\mathbf {fgfMod}_R}\,}}\rightarrow {{\,\mathrm{\mathbf {fgMod}_R}\,}}$ of finite degree.

For any $U,V \in {{\,\mathrm{\mathbf {fgfMod}_R}\,}}$ we have a sequence of polynomial lawswhose composition we denote by $\Phi _{U,V}$. We also let $\Pi _{U,V}:{{\,\mathrm{Hom}\,}}(U,V) \times P(U)\rightarrow P(U)$ be the linear polynomial law given by projection. Recall that $\Phi _{U,V}$ and $\Pi _{U,V}$ both yield continuous maps from ${{\,\mathrm{{\mathbb A}}\,}}_{{{\,\mathrm{Hom}\,}}(U,V) \times P(U)}\rightarrow {{\,\mathrm{{\mathbb A}}\,}}_{P(V)}$.

#### Definition 61

We define ${{\,\mathrm{{\mathbb A}}\,}}_P$ to be *P*. A *subset* of ${{\,\mathrm{{\mathbb A}}\,}}_P$ is a rule *X* that assigns to each $U \in {{\,\mathrm{\mathbf {fgfMod}_R}\,}}$ a subset *X*(*U*) of ${{\,\mathrm{{\mathbb A}}\,}}_{P(U)}$ (see Definition 30) in such a manner that

$$\begin{aligned} \Phi _{U,V}(\Pi _{U,V}^{-1}(X(U))) \subseteq X(V) \end{aligned}$$

for all $U,V \in {{\,\mathrm{\mathbf {fgfMod}_R}\,}}$. The subset $X\subseteq {{\,\mathrm{{\mathbb A}}\,}}_P$ is *closed* if *X*(*U*) is a closed subset of ${{\,\mathrm{{\mathbb A}}\,}}_{P(U)}$ for all $U \in {{\,\mathrm{\mathbf {fgfMod}_R}\,}}$. The *closure* of *X* is the closed subset $\overline{X}$ of ${{\,\mathrm{{\mathbb A}}\,}}_P$ assigning $\overline{X(U)}$ to *U* for all $U\in {{\,\mathrm{\mathbf {fgfMod}_R}\,}}$.

It is worth spelling out what this means. Let *U*, *V* be finitely generated free *R*-modules, let *D* be an *R*-domain and let $\varphi \in D \otimes {{\,\mathrm{Hom}\,}}(U,V)$. Then the condition is that $P_{U,V,D}(\varphi )\in D\otimes {{\,\mathrm{Hom}\,}}(P(U),P(V))$ maps $X(U)(D)\subseteq D\otimes P(U)$ into *X*(*V*)(*D*). In the particular case where $V=U$, this condition can be informally thought of as the condition that *X*(*U*) is preserved under the polynomial action of ${{\,\mathrm{End}\,}}(U)$. Let $\alpha :Q\rightarrow P$ be a polynomial transformation and let *X* be a subset of *Q*. Then $\alpha (X)=(U\mapsto \alpha _U(X(U)))$ is a subset of *P*.

#### Definition 62

For $X\subseteq {{\,\mathrm{{\mathbb A}}\,}}_P$, we define the ideal ${{\,\mathrm{\mathcal {I}}\,}}_X$ of *X* to be the rule assigning ${{\,\mathrm{\mathcal {I}}\,}}_{X(U)} \subseteq R[P(U)]$ to *U* for all $U\in {{\,\mathrm{\mathbf {fgfMod}_R}\,}}$. The rule ${{\,\mathrm{\mathcal {I}}\,}}_X$ is an ideal in the *R*-algebra over the category ${{\,\mathrm{\mathbf {fgfMod}_R}\,}}$ defined by $U \mapsto R[P(U)]$, i.e., for all $\varphi \in {{\,\mathrm{Hom}\,}}(U,V)$ we have ${{\,\mathrm{\mathcal {I}}\,}}_X(V)\circ P_{U,V,R}(\varphi )\subseteq {{\,\mathrm{\mathcal {I}}\,}}_X(U)$.

#### Definition 63

(*Base change*). If $X\subseteq {{\,\mathrm{{\mathbb A}}\,}}_P$ is a closed subset and *B* is an *R*-algebra, then we obtain a closed subset $X_B$ of ${{\,\mathrm{{\mathbb A}}\,}}_{P_B}$ by letting, for a $U \in {{\,\mathrm{\mathbf {fgfMod}_B}\,}}$, $X_B(U)$ be the closed subset $X(U_R)_B$ of ${{\,\mathrm{{\mathbb A}}\,}}_{P_B(U)}={{\,\mathrm{{\mathbb A}}\,}}_{B \otimes P(U_R)}$, where $U_R$ is the free *R*-module such that $U \cong B \otimes U_R$ from the definition of $P_B$.

We will use the following lemmas very frequently in our proof of Theorem 2.

#### Lemma 64

Let *R* be a ring with Noetherian spectrum and *r* an element of *R*. Let $\mathfrak {p}_1,\ldots ,\mathfrak {p}_k$ be the minimal primes of *R*/(*r*). Then two closed subsets $X,Y\subseteq {{\,\mathrm{{\mathbb A}}\,}}_P$ are equal if and only if $X_{R[1/r]}=Y_{R[1/r]}$ and $X_{R/\mathfrak {p}_i}=Y_{R/\mathfrak {p}_i}$ for all $i=1,\ldots ,k$.

#### Proof

This follows from Lemma 37 with *X*(*U*), *Y*(*U*) for every $U\in {{\,\mathrm{\mathbf {fgfMod}_R}\,}}$. $\square$

#### Lemma 65

Let $R\subseteq R'$ be a finite extension of domains and let $X,Y\subseteq {{\,\mathrm{{\mathbb A}}\,}}_P$ be closed subsets. Then $X=Y$ if and only if $X_{R'}=Y_{R'}$.

#### Proof

This follows from Lemma 38 with *X*(*U*), *Y*(*U*) for every $U\in {{\,\mathrm{\mathbf {fgfMod}_R}\,}}$. $\square$

#### Lemma 66

Let $U\in {{\,\mathrm{\mathbf {fgfMod}_R}\,}}$ and $g\in R[P(U)]$. Then

$$\begin{aligned} Y(V)(D)=\{p\in D\otimes P(V)\mid \forall \varphi \in D\otimes {{\,\mathrm{Hom}\,}}(V,U): g_D(P_{V,U,D}(\varphi )(p))=0\} \end{aligned}$$

for all $V\in {{\,\mathrm{\mathbf {fgfMod}_R}\,}}$ and *R*-domains *D* defines a closed subset $Y\subseteq {{\,\mathrm{{\mathbb A}}\,}}_P$. The subset *Y* is the biggest closed subset of ${{\,\mathrm{{\mathbb A}}\,}}_P$ such that *g* is in the ideal of *Y*(*U*).

#### Proof

It is easy to check that *Y*(*V*) is a subset of ${{\,\mathrm{{\mathbb A}}\,}}_{P(V)}$ for all $V\in {{\,\mathrm{\mathbf {fgfMod}_R}\,}}$ and that *Y* is a subset of ${{\,\mathrm{{\mathbb A}}\,}}_P$. We need to check that *Y* is a closed subset of ${{\,\mathrm{{\mathbb A}}\,}}_P$, i.e., that *Y*(*V*) is a closed subset of ${{\,\mathrm{{\mathbb A}}\,}}_{P(V)}$ for every $V\in {{\,\mathrm{\mathbf {fgfMod}_R}\,}}$.

Let $\varphi _1,\ldots ,\varphi _n$ be a basis of ${{\,\mathrm{Hom}\,}}(V,U)$. For every *R*-algebra *A*, consider the map

$$\begin{aligned}&g_{A[x_1,\ldots ,x_n]}\circ P_{V,U,A[x_1,\ldots ,x_n]}(x_1\otimes \varphi _1+\cdots +x_n\otimes \varphi _n)\\&\quad :A[x_1,\ldots ,x_n]\otimes P(V)\rightarrow A[x_1,\ldots ,x_n]. \end{aligned}$$

We have

$$\begin{aligned} g_{A[x_1,\ldots ,x_n]}\circ P_{V,U,A[x_1,\ldots ,x_n]}(x_1\otimes \varphi _1+\cdots +x_n\otimes \varphi _n)|_{A\otimes P(V)}=\sum _{\alpha \in {{\,\mathrm{{\mathbb Z}}\,}}_{\ge 0}^n} x^{\alpha }g_{\alpha ,A} \end{aligned}$$

where $g_{\alpha ,A}:A\otimes P(V)\rightarrow A$. We get polynomial laws $g_{\alpha }=(g_{\alpha ,A})_A\in R[P(V)]$. Set $S_V=\{g_\alpha \mid \alpha \in {{\,\mathrm{{\mathbb Z}}\,}}_{\ge 0}^n\}$. We claim that $Y(V)={{\,\mathrm{\mathcal {V}}\,}}(S_V)$. Let *D* be an *R*-domain and take $p\in Y(V)(D)$. Then, viewing *p* as an element of $Y(V)(D[x_1,\ldots ,x_n])$, we see that

$$\begin{aligned} g_{D[x_1,\ldots ,x_n]}(P_{V,U,D[x_1,\ldots ,x_n]}(\varphi )(p))=0 \end{aligned}$$

for all $\varphi \in D[x_1,\ldots ,x_n]\otimes {{\,\mathrm{Hom}\,}}(V,U)$. Using $\varphi =x_1\otimes \varphi _1+\cdots + x_n\otimes \varphi _n$, we get $p\in {{\,\mathrm{\mathcal {V}}\,}}(S_V)(D)$. Conversely, suppose that $p\in {{\,\mathrm{\mathcal {V}}\,}}(S_V)(D)$. Then

$$\begin{aligned} g_{D[x_1,\ldots ,x_n]}(P_{V,U,D[x_1,\ldots ,x_n]}(x_1\otimes \varphi _1+\cdots +x_n\otimes \varphi _n)(p))=0 \end{aligned}$$

Specializing the $x_i$ to elements of *D*, we find that

$$\begin{aligned} g_D(P_{V,U,D}(a_1\otimes \varphi _1+\cdots +a_n\otimes \varphi _n)(p))=0 \end{aligned}$$

for all $a_1,\ldots ,a_n\in D$. So $p\in Y(V)(D)$. So $Y(V)={{\,\mathrm{\mathcal {V}}\,}}(S_V)$ is indeed closed. $\square$

#### Remark 67

It is not true in general that

$$\begin{aligned} Y(V)(D) = \{p \in X(V)(D) \mid \forall \varphi \in {{\,\mathrm{Hom}\,}}(V, U): h_D(P(\varphi )_D(p)) = 0 \}. \end{aligned}$$

For an example, take $R={{\,\mathrm{{\mathbb F}}\,}}_p$, $P(V)=V$ and $h=x^p-x\in R[x]=R[P(R)]$. Then the right hand side above consists of all $p\in D\otimes V\cong D^n$ such that $x^p=x$ for every coordinate of *p* while the left hand side also has the requirement that $(\alpha x)^p=\alpha x$ for all $\alpha \in E$ for every *D*-domain *E*. So $Y(V)(D)=0$.

### Gradings

Let $P:{{\,\mathrm{\mathbf {fgfMod}_R}\,}}\rightarrow {{\,\mathrm{\mathbf {fgMod}_R}\,}}$ be a polynomial functor. For each $U \in {{\,\mathrm{\mathbf {fgfMod}_R}\,}}$, the *R*-algebra *R*[*P*(*U*)] has two natural gradings: first, the *ordinary* grading that each coordinate ring *R*[*M*] of a module *M* has (see Definition 19); and second, a grading that takes into account the degrees of the homogeneous components *P*, as follows. Write $P=P_0 \oplus P_1 \oplus \cdots \oplus P_d$, so that *R*[*P*(*U*)] is the tensor product of the $R[P_i(U)]$ by Proposition 27. Then multiply the ordinary grading on $R[P_i(U)]$ by *i* and use these to define a grading on *R*[*P*(*U*)], called the *standard* grading. The standard grading has an alternative characterisation, as follows: $f \in R[P(U)]$ is homogeneous of degree *j* if $f_A( P_{U,U,A}(a \otimes {{\,\mathrm{id}\,}}_U)(v))=a^j f_A(v)$ for all $A \in {{\,\mathrm{\mathbf {Alg}_R}\,}}$ and all $v \in A \otimes P(U)$. We have

$$\begin{aligned} f_{A[t]}(v_0+tv_1+\cdots +t^dv_d)=\sum _{j=0}^{\infty }t^j f_{j,A}(v_0+v_1+\cdots +v_d) \end{aligned}$$

for all $A\in {{\,\mathrm{\mathbf {Alg}_R}\,}}$ and $v_i\in A\otimes P_i(U)$ where $f_j$ is the part of *f* of standard degree *j*.

#### Lemma 68

For any closed subset $X\subseteq {{\,\mathrm{{\mathbb A}}\,}}_P$ and any $U \in {{\,\mathrm{\mathbf {fgfMod}_R}\,}}$, the ideal ${{\,\mathrm{\mathcal {I}}\,}}_X(U)$ is homogeneous with respect to the standard grading.

#### Proof

Take $f\in {{\,\mathrm{\mathcal {I}}\,}}_X(U)$ and let *D* be an *R*-domain. Then

$$\begin{aligned} 0=f_{D[t]}(P_{U,U,D[t]}(t\otimes {{\,\mathrm{id}\,}}_{U})(v_0+v_1+\cdots +v_d))=f_{D[t]}(v_0+tv_1+\cdots +t^dv_d) \end{aligned}$$

for all $v_i\in D\otimes P_i(U)$ such that $v_0+v_1+\cdots +v_d\in X(U)(D)$. Hence the homogeneous parts of *f* are also contained in ${{\,\mathrm{\mathcal {I}}\,}}_X(U)$. $\square$

## Proof of the main theorem

In this section we prove Theorem 2. Let *R* be a ring whose spectrum is Noetherian and let $P:{{\,\mathrm{\mathbf {fgfMod}_R}\,}}\rightarrow {{\,\mathrm{\mathbf {fgMod}_R}\,}}$ a polynomial functor of finite degree. We will prove that any chain ${{\,\mathrm{{\mathbb A}}\,}}_P \supseteq X_1 \supseteq X_2 \supseteq \cdots$ of closed subsets eventually stabilises.

### Reduction to the case of a domain

Since ${{\,\mathrm{Spec}\,}}(R)$ is Noetherian, the ring *R* has finitely many minimal primes $\mathfrak {p}_1,\ldots ,\mathfrak {p}_k$. By Lemma 64 with $r=1$, the sequence ${{\,\mathrm{{\mathbb A}}\,}}_P\supseteq X_1 \supseteq X_2 \supseteq \cdots$ stabilises if and only if the sequence ${{\,\mathrm{{\mathbb A}}\,}}_{P_{R/\mathfrak {p}_i}}\supseteq X_{1,R/\mathfrak {p}_i} \supseteq X_{2,R/\mathfrak {p}_i} \supseteq \cdots$ stabilises for each $i\in [k]$. So from now on we assume that *R* is a domain, we write $K_\mathfrak {p}:={{\,\mathrm{Frac}\,}}(R/\mathfrak {p})$ for $\mathfrak {p}\in {{\,\mathrm{Spec}\,}}(R)$, $K:=K_{(0)}={{\,\mathrm{Frac}\,}}(R)$, and we let $\overline{K},\overline{K_\mathfrak {p}}$ be algebraic closures of $K,K_\mathfrak {p}$, respectively.

### A stronger statement

We will prove the following stronger statement which clearly implies Theorem 2.

#### Theorem 69

Let (*R*, *P*, *X*) be a triple consisting of a domain *R* with Noetherian spectrum, a polynomial functor $P:{{\,\mathrm{\mathbf {fgfMod}_R}\,}}\rightarrow {{\,\mathrm{\mathbf {fgMod}_R}\,}}$ of finite degree and a closed subset $X\subseteq {{\,\mathrm{{\mathbb A}}\,}}_P$. Then (*R*, *P*, *X*) satisfies the following conditions:

1. Every descending chain $X=X_1 \supseteq X_2\supseteq \cdots$ of closed subsets of *X* eventually stabilises.
2. There exists a nonzero $r \in R$ such that the following holds for all $U \in {{\,\mathrm{\mathbf {fgfMod}_R}\,}}$: if $f \in R[P(U)]$ vanishes identically on $X(U)(\overline{K})$, then *f* vanishes identically on $X(U)(\overline{K_\mathfrak {p}})$ for all primes $\mathfrak {p}\in {{\,\mathrm{Spec}\,}}(R[1/r])$.

#### Remark 70

Condition (2) of the theorem means that ${{\,\mathrm{\mathcal {I}}\,}}_{X_{R[1/r]}}$ is determined by ${{\,\mathrm{\mathcal {I}}\,}}_{X_{\overline{K}}}$. More precisely, setting $R'=R[1/r]$, for every $U\in {{\,\mathrm{\mathbf {fgfMod}}\,}}_{R'}$, the ideal

$$\begin{aligned} {{\,\mathrm{\mathcal {I}}\,}}_{X_{R'}}(U)={{\,\mathrm{\mathcal {I}}\,}}_{X_{R'}(U)}\subseteq R'[P_{R'}(U)] \end{aligned}$$

is the pull-back of the ideal in $\overline{K}[P_{R'}(\overline{K}\otimes U)]$ of the affine variety $X_{R'}(\overline{K}\otimes U)$.

The proof of Theorem 69 is a somewhat intricate induction, combining induction on *P*, Noetherian induction on ${{\,\mathrm{Spec}\,}}(R)$ and induction on minimal degrees of functions in the ideal of *X*—for details, see below.

#### Notation 71

For any fixed triple (*R*, *P*, *X*), we denote conditions (1) and (2) of Theorem 69 by $\Sigma (R, P, X)$.

### The induction base

If *P* has degree zero, then *X* is just a closed subset of ${{\,\mathrm{{\mathbb A}}\,}}_{P(0)}$. Here, the Noetherianity statement is Proposition 1 and the statement about vanishing functions is Proposition 44.

### The outer induction

To prove the theorem for *P* of positive degree, we will show that $\Sigma (R,P,X)$ is implied by $\Sigma (R',P',X')$ where $X'$ is a closed subset of ${{\,\mathrm{{\mathbb A}}\,}}_{P'}$ and $(R',P')$ ranges over pairs that have one of the following forms:

(i) $(R',P')=(R/\mathfrak {p},P_{R/\mathfrak {p}})$ for some nonzero prime $\mathfrak {p}$ of *R*; or
(ii) $(R',P')$ where $R'$ is a domain that is a finite extension of a localisation *R*[1/*r*] of *R*, $\deg P' \le \deg P=:d$, for $K':={{\,\mathrm{Frac}\,}}(R')$ we have $P'_{K'} \not \cong P_{K'}$ and for the largest *e* such that the homogeneous parts $P'_{e,K'}$ and $P_{e,K'}$ are not isomorphic, the former is a quotient of the latter.

In both cases, we write $(R,P) \rightarrow (R',P')$. We consider the class $\Pi$ of all the pairs (*R*, *P*). The reflexive and transitive closure of the relation $\rightarrow$ is a partial order on $\Pi$.

#### Lemma 72

The partial order on $\Pi$ is well-founded.

#### Proof

Suppose that we had an infinite sequence

$$\begin{aligned} (R_0,P_0)\rightarrow (R_1,P_1) \rightarrow (R_2,P_2) \rightarrow \cdots \end{aligned}$$

of such steps. By the Friedlander–Suslin lemma, any sequence of steps of type (ii) only must terminate (see also [8, Lemma 12]). So our sequence contains infinitely many steps of type (i).

Each step $(R,P) \rightarrow (R',P')$ induces a morphism $\alpha :{{\,\mathrm{Spec}\,}}(R') \rightarrow {{\,\mathrm{Spec}\,}}(R)$. This morphism $\alpha$ has the property that for irreducible closed subsets $C \subsetneq D\subseteq {{\,\mathrm{Spec}\,}}(R')$, we have $\overline{\alpha (C)}\subsetneq \overline{\alpha (D)}$. This holds trivially for steps of type (i), where the morphism $\alpha :{{\,\mathrm{Spec}\,}}(R/\mathfrak {p})\rightarrow {{\,\mathrm{Spec}\,}}(R)$ is a closed embedding, and also for steps of type (ii) by elementary properties of localisation and of integral extensions of rings (see, e.g., [10, Corollary 4.18 (Incomparability)]).

Let $\alpha _i:{{\,\mathrm{Spec}\,}}(R_i)\rightarrow {{\,\mathrm{Spec}\,}}(R_{i-1})$ be the morphism induced by $(R_{i-1},P_{i-1}) \rightarrow (R_i,P_i)$ and take $\beta _i=\alpha _1 \circ \cdots \circ \alpha _i:{{\,\mathrm{Spec}\,}}(R_i) \rightarrow {{\,\mathrm{Spec}\,}}(R_0)$. Then the maps $\beta _i$ have the same incomparability property as the $\alpha _i$. Hence, whenever the step $(R_{i-1},P_{i-1}) \rightarrow (R_{i},P_{i})$ is of type (i), there is the inclusion of irreducible closed sets ${{\,\mathrm{im}\,}}\alpha _i \subsetneq {{\,\mathrm{Spec}\,}}(R_{i-1})$ and therefore $\overline{{{\,\mathrm{im}\,}}\beta _i}\subsetneq \overline{{{\,\mathrm{im}\,}}\beta _{i-1}}$ is a strict inclusion. This contradicts the Noetherianity of ${{\,\mathrm{Spec}\,}}(R_0)$. $\square$

By Lemma 72 we can proceed by induction on $\Pi$, namely, in proving that $\Sigma (R,P,X)$ holds, we may assume $\Sigma (R', P', X')$ whenever $(R',P')\leftarrow (R,P)$.

#### Lemma 73

Let $r\in R$ be a nonzero element and let $\mathfrak {p}_1,\ldots ,\mathfrak {p}_k$ be the minimal primes of *R*/(*r*). Assume that $\Sigma (R[1/r],P_{R[1/r]},X_{R[1/r]})$ and $\Sigma (R/\mathfrak {p}_i,P_{R/\mathfrak {p}_i},X_{R/\mathfrak {p}_i})$ for each $i\in [k]$ hold. Then $\Sigma (R,P,X)$ holds as well.

#### Proof

By Lemma 64, we see that condition (1) for (*R*, *P*, *X*) follows from condition (1) for $(R[1/r],P_{R[1/r]},X_{R[1/r]})$ together with $\Sigma (R/\mathfrak {p}_i,P_{R/\mathfrak {p}_i},X_{R/\mathfrak {p}_i})$ for each $i\in [k]$. Condition (2) for (*R*, *P*, *X*) follows from condition (2) for $(R[1/r],P_{R[1/r]},X_{R[1/r]})$. $\square$

Combining this lemma with our induction hypothesis, we see that in order to prove $\Sigma (R,P,X)$ it suffices to prove $\Sigma (R[1/r],P_{R[1/r]},X_{R[1/r]})$ for some $r\in R$. So we may replace (*R*, *P*, *X*) by $(R[1/r],P_{R[1/r]},X_{R[1/r]})$ whenever this is convenient.

### Finding an irreducible factor

Now let $P:{{\,\mathrm{\mathbf {fgfMod}_R}\,}}\rightarrow {{\,\mathrm{\mathbf {fgMod}_R}\,}}$ be a fixed polynomial functor of degree $d>0$ over a domain *R* with Noetherian spectrum. Recall that *K* is the fraction field of *R*.

Suppose first that the base change $P_{K}$ has degree $<d$. Then $K \otimes P_d(U)=0$ for all $U \in {{\,\mathrm{\mathbf {fgfMod}_R}\,}}$. In particular, this holds for $U=R^d$. So since $P_d(U)$ is a finitely generated *R*-module, there exists a nonzero $r \in R$ such that $R[1/r] \otimes P_d(U)=0$. By the Friedlander–Suslin lemma (Theorem 57), we then find $(P_d)_{R[1/r]}=0$. In this case, we replace (*R*, *P*, *X*) by $(R[1/r],P_{R[1/r]},X_{R[1/r]})$. By repeating this at most *d* times, we may assume that the base change $P_{K}$ has the same degree as *P*.

We want a polynomial subfunctor *M* of the top-degree part $P_d$ of *P* whose base change with $\overline{K}$ is an irreducible polynomial subfunctor of $(P_d)_{\overline{K}}$. In the next lemma, we show that such an *M* exists after passing from *R* to a suitable finite extension of one of its localisations.

#### Proposition 74

There exist a finite extension $R'$ of a localisation *R*[1/*r*] of *R* and a polynomial subfunctor *M* of the top-degree part of the polynomial functor $P_{R'}$ such that the base change $M_{\overline{K}}$ is an irreducible polynomial subfunctor of $P_{d,\overline{K}}$.

#### Proof

The $S_d(\overline{K}^d)$-module $P_{d,\overline{K}}(\overline{K}^d) = \overline{K}\otimes P_d(R^d)$ is finite-dimensional and hence has an irreducible submodule $N'$. It is finitely generated, say of dimension $n>0$. Let $\sum _j\alpha _{ij} \otimes m_{ij}$ for $i = 1, \ldots , n$ be a $\overline{K}$-basis. By the Friedlander–Suslin lemma, the irreducible submodule $N'$ corresponds to an irreducible polynomial subfunctor *N* of $P_{d,\overline{K}}$. The elements $\alpha _i$ are algebraic over the fraction field *K* of *R*. Let $r\in R$ be the product of all the denominators appearing in their minimal polynomials. Then $R' = R[1/r][\alpha _1, \ldots , \alpha _n]$ is a finite extension of the localisation *R*[1/*r*] of *R* since the $\alpha _i$ are integral over *R*[1/*r*]. Consider the submodule $M'$ of the $S_d(R'^d)$-module $P_{d,R'}((R')^d)$ generated by the elements $\sum _j\alpha _{ij} \otimes m_{ij}$. By the Friedlander–Suslin lemma, $M'$ corresponds to a polynomial subfunctor *M* of $P_{d,R'}$ whose base change $M_{\overline{K}}=N$ is an irreducible polynomial subfunctor of $P_{d,\overline{K}}$. $\square$

Let $r\in R$ and $R'$ be as in the previous proposition. We would like to reduce to the case where $R'=R$. As before, we can replace (*R*, *P*, *X*) by $(R[1/r],P_{R[1/r]},X_{R[1/r]})$, so that $R'$ is a finite extension of *R*. We now prove a version of Lemma 73 for such extensions.

#### Lemma 75

Assume that $\Sigma (R',P_{R'},X_{R'})$ holds. Then $\Sigma (R,P,X)$ holds as well.

#### Proof

By Lemma 65, condition (1) for $(R',P_{R'},X_{R'})$ implies condition (1) for (*R*, *P*, *X*). Let $r' \in R'$ be a nonzero element as in condition (2) for $(R',P_{R'},X_{R'})$, i.e., for every $U\in {{\,\mathrm{\mathbf {fgfMod}}\,}}_{R'}$, every $f\in R'[P_{R'}(U)]$ vanishing identically on $X_{R'}(U)(\overline{K})$ also vanishes identically on $X_{R'}(U)(\overline{K_{\mathfrak {p}}})$ for every prime ideal $\mathfrak {p}\in {{\,\mathrm{Spec}\,}}(R'[1/r'])$. Now $(r')\cap R$ is not the zero ideal, since $r'$ is nonzero and integral over *R*. Pick any nonzero $r \in (r') \cap R$. We claim that condition (2) holds for (*R*, *P*, *X*) with this particular *r*.

Indeed, let $U_R\in {{\,\mathrm{\mathbf {fgfMod}_R}\,}}$ and take $U:=R'\otimes U_R$. Let *f* be an element of $R[P(U_R)]$ vanishing identically on $X(U_R)(\overline{K})$. Then *f* is naturally induces an element of $R'[P_{R'}(U)]$ vanishing identically on $X_{R'}(U)(\overline{K})=X(U_R)(\overline{K})$. So we see that *f* vanishes on $X_{R'}(U)(\overline{K_{\mathfrak {q}}})$ for each $\mathfrak {q}\in {{\,\mathrm{Spec}\,}}(R'[1/r'])$. Since $R'$ is integral over *R*, for any $\mathfrak {p}\in {{\,\mathrm{Spec}\,}}(R)$ there exists an $\mathfrak {q}\in {{\,\mathrm{Spec}\,}}(R')$ with $\mathfrak {q}\cap R=\mathfrak {p}$; and if, moverover, the prime ideal $\mathfrak {p}$ does not contain *r*, then the prime ideal $\mathfrak {q}$ does not contain $r'$. Hence *f* vanishes identically on $\overline{K_{\mathfrak {p}}}$, as desired. $\square$

We replace (*R*, *P*, *X*) by $(R',P_{R'},X_{R'})$, so that there exists a polynomial subfunctor *M* of the top-degree part $P_d$ of *P* such that the base change $M_{\overline{K}}$ is an irreducible polynomial subfunctor of $P_{d,\overline{K}}$.

### Splitting off *M*

Proposition 55 guarantees that after passing to a further localisation (and using Noetherian induction for the complement), we may assume that for each $U \in {{\,\mathrm{\mathbf {fgfMod}_R}\,}}$, the *R*-module *P*(*U*) is the direct sum of a finitely generated free *R*-module and the (also finitely generated free) *R*-module *M*(*U*). In particular, both *P* and $P':=P/M$ are polynomial functors ${{\,\mathrm{\mathbf {fgfMod}_R}\,}}\rightarrow {{\,\mathrm{\mathbf {fgfMod}_R}\,}}$.

Let $\pi :P\rightarrow P'$ be the projection morphism. For a closed subset $X\subseteq {{\,\mathrm{{\mathbb A}}\,}}_P$, we define the closed subset $X'\subseteq {{\,\mathrm{{\mathbb A}}\,}}_{P'}$ as the closure of $\pi (X)$. Note that $(R,P)\rightarrow (R,P')$ and hence $\Sigma (R,P',X')$ holds. In particular, we may and will replace *R* by a further localisation *R*[1/*r*] which ensures that, if $f \in R[P'(U)]$ vanishes identically on $X'(U)(\overline{K})$, then it vanishes identically on $X'(U)(\overline{K_{\mathfrak {p}}})$ for all $\mathfrak {p}\in {{\,\mathrm{Spec}\,}}(R)$.

### The inner induction

We perform the same inner induction as in [8, §2.9]. Let $\delta _X \in \{0,1,\ldots ,\infty \}$ denote the smallest degree, in the standard grading, of a homogeneous element of $R[P(U)] \cong R[M(U)] \otimes R[P'(U)]$ (here we use that *P*(*U*) is the direct sum of the *R*-modules *M*(*U*) and $P'(U)$), over all $U \in {{\,\mathrm{\mathbf {fgfMod}_R}\,}}$, that lies in the vanishing ideal of *X*(*U*) but does not lie in the vanishing ideal of the pre-image in ${{\,\mathrm{{\mathbb A}}\,}}_{P(U)}$ of $X'(U) \subseteq {{\,\mathrm{{\mathbb A}}\,}}_{P'(U)}$. Note that $\delta _X=0$ is, in fact, impossible, since the coordinates on *R*[*M*(*U*)] have positive degree, so that a degree-0 homogeneous element of *R*[*P*(*U*)] that lies in the ideal of *X*(*U*) is an element of $R[P'(U)]$ that lies in the ideal of $X'(U)$. At the other extreme, $\delta _X=\infty$ means that *X*(*U*) is the Cartesian product of $X'(U)$ with ${{\,\mathrm{{\mathbb A}}\,}}_{M(U)}$ for all *U*. We order closed subsets of ${{\,\mathrm{{\mathbb A}}\,}}_P$ by $Y<X$ if either $Y' \subsetneq X'$ or else $Y'=X'$ but $\delta _Y<\delta _X$. Note that, by the outer induction hypothesis for $\Sigma (R, P', X')$ and since $\{0,1,\ldots ,\infty \}$ is well-ordered, this order is well-founded. Hence when proving $\Sigma (P,R,X)$, we may assume that $\Sigma (P,R,Y)$ holds for all $Y<X$.

First suppose that $\delta _X=\infty$. Then, for all proper closed subsets *Y* of *X*, we have $Y<X$ and so $\Sigma (R,P,Y)$ holds by the inner induction hypothesis. It follows that condition (1) holds for (*R*, *P*, *X*). Condition (2) for (*R*, *P*, *X*) follows from condition (2) for $(R,P',X')$, with the same $r \in R$ to be inverted. Indeed, if $f\in R[P(U)]\cong R[M(U)] \otimes R[P'(U)]$ vanishes identically $X(U)(\overline{K})\cong {{\,\mathrm{{\mathbb A}}\,}}_{M(U)}(\overline{K})\times X'(U)(\overline{K})$, then, regarding *f* as a polynomial in the coordinates on *M*(*U*) with coefficients in $R[P'(U)]$, those coefficients must all vanish identically on $X'(U)(\overline{K})$, hence on $X'(U)(\overline{K_\mathfrak {p}})$ for all $\mathfrak {p}\in {{\,\mathrm{Spec}\,}}(R[1/r])$.

### A directional derivative

Next, suppose that $1 \le \delta _X<\infty$. Let $f \in R[P(U)]\cong R[M(U)] \otimes R[P'(U)]$ be a homogeneous polynomial of degree $\delta _X$ in the standard grading, which lies in the ideal of *X*(*U*) but not on the preimage in ${{\,\mathrm{{\mathbb A}}\,}}_{P(U)}$ of $X'(U)$. Expanding *f* as a polynomial in the coordinates on *R*[*M*(*U*)] with coefficients in $R[P'(U)]$, one of those coefficients does not lie in the ideal of $X'(U)$. Our assumptions together with Corollary 36 guarantee that, in fact, that coefficient does not vanish identically on $X'(U)(\overline{K})$, so that *f* does not vanish identically on the pre-image of $X'(U)(\overline{K})$ in ${{\,\mathrm{{\mathbb A}}\,}}_{P(U)}(\overline{K})$. We then proceed as in [8, Lemma 18]. Let $v_1, \ldots , v_m$ be an *R*-basis of *M*(*U*) and extend this with $v_{m+1}, \ldots , v_n$ to an *R*-basis of *P*(*U*), inducing an isomorphism $R[P(U)]\cong R[x_1,\ldots ,x_n]$. The expression

$$\begin{aligned} f_{R[x_1,\ldots ,x_n,y_1,\ldots ,y_m,t]}\left( \sum _{i=1}^n x_i \otimes v_i + \sum _{j=1}^m ty_j \otimes v_j\right) \in R[x_1,\ldots ,x_n,y_1,\ldots ,y_m,t] \end{aligned}$$

explicitly reads as

$$\begin{aligned} f(x_1+t y_1, x_2+t y_2,\ldots ,x_m+t y_m, x_{m+1},\ldots ,x_n). \end{aligned}$$

Take $p=1$ if ${{\,\mathrm{char}\,}}R=0$ and $p={{\,\mathrm{char}\,}}R$ otherwise. A Taylor expansion in *t* turns this expression into

$$\begin{aligned} f(x_1,\ldots ,x_n) + t^{p^e}\cdot \left( h_1(x_1,\ldots ,x_n) y_1^{p^e}+\cdots +h_m(x_1,\ldots ,x_n) y_m^{p^e}\right) + t^{p^e+1}\cdot g \end{aligned}$$

for some integer $e\ge 0$, polynomial $g\in R[x_1,\ldots ,x_n,y_1,\ldots ,y_m,t]$ and homogeneous polynomials $h_i \in R[P(U)]$ of (standard) degree $\delta _X-p^e d$ not all vanishing identically on $X(U)(\overline{K})$. Specialising the variables $y_i$ to values $a_i\in \{0,1\}$, we get that

$$\begin{aligned} h(x_1,\ldots ,x_n):=\sum _{i=1}^m a_i^{p^e}h_i(x_1,\ldots ,x_n) \in R[P(U)] \end{aligned}$$

does not vanish identically on $X(U)(\overline{K})$.

Let $p\in \overline{K}\otimes P(U)$ be a point in $X(U)(\overline{K})$ such that $h_{\overline{K}}(p)\ne 0$. Relative to the chosen basis of *P*(*U*), we may write $p = (\alpha _1,\ldots ,\alpha _n)$. Reasoning as before, let $r \in R$ be the product of all the denominators appearing in the minimal polynomials of the $\alpha _i$ over *K* so that $R' = R[1/r][\alpha _1, \cdots , \alpha _k]$ is a finite extension of *R*[1/*r*] containing all $\alpha _i$. Replacing *R* by $R'$ and using Lemma 75, we can therefore assume that $p\in X(U)(R)$ satisfies $h_R(p)\ne 0$. Further replacing *R* by $R[1/h_R(p)]$, we find that $h_D(p)\ne 0$ for all *R*-domains *D*. Define *Y* to be the biggest closed subset of *X* where *h* does vanish.

#### Lemma 76

We have

$$\begin{aligned} Y(V)(D)=\{p\in X(V)(D)\mid \forall \varphi \in D\otimes {{\,\mathrm{Hom}\,}}(V,U): h_D(P_{V,U,D}(\varphi )(p))=0\} \end{aligned}$$

for all $V\in {{\,\mathrm{\mathbf {fgfMod}_R}\,}}$ and *R*-domains *D*.

#### Proof

The closed subset *Y* is the intersection of *X* with the biggest closed subset of ${{\,\mathrm{{\mathbb A}}\,}}_P$ where *h* vanishes. So the lemma follows from Lemma 66. $\square$

Let $X=X_1 \supseteq X_2 \supseteq \cdots$ be a sequence of closed subsets of *X*. Since $Y<X$, the statement $\Sigma (R,P,Y)$ holds by the inner induction. In particular, the intersections of the $X_i$ with *Y* stabilise. This settles part of condition (1) of $\Sigma (R,P,X)$. We now develop the theory to deal with the complement of *Y*. This will afterwards be used to settle both condition (2) for $\Sigma (R,P,X)$ in Sect. 6.10 and complete the proof of condition (1) in Sect. 6.11.

### Dealing with the localised shift

In [8, Lemma 25], it is proved that for all $\mathfrak {p}\in {{\,\mathrm{Spec}\,}}(R)$ and $V \in {{\,\mathrm{\mathbf {fgfMod}_R}\,}}$, the projection ${{\,\mathrm{Sh}\,}}_U(P)\rightarrow {{\,\mathrm{Sh}\,}}_U(P)/M$ induces a homeomorphism of ${{\,\mathrm{Sh}\,}}_U(X)[1/h](V)(\overline{K_\mathfrak {p}})$ with a closed subset of the basic open $({{\,\mathrm{Sh}\,}}_U(P)/M)[1/h](V)(\overline{K_{\mathfrak {p}}})$. This proof uses that $M_{\overline{K}_\mathfrak {p}}$ is irreducible, which is why we have localised so as to make this true. The proof shows that, indeed, for each linear function $x \in (\overline{K_{\mathfrak {p}}} \otimes M(V))^*$, the $p^e$-th power $x^{p^e}$ lies in the sum of the ideal of ${{\,\mathrm{Sh}\,}}_U(X)[1/h](V)(\overline{K_\mathfrak {p}})$ in $\overline{K_\mathfrak {p}}[\overline{K_{\mathfrak {p}}}\otimes P(U \oplus V)][1/h]$ and the subring $\overline{K_\mathfrak {p}}[\overline{K_{\mathfrak {p}}}\otimes (P(U \oplus V)/M(V))]$. We globalise this result as follows: for all $V \in {{\,\mathrm{\mathbf {fgfMod}_R}\,}}$, define

$$\begin{aligned} N(V):=\left\{ x \in M(V)^* \,\bigg |\, x^{p^e} \in {{\,\mathrm{\mathcal {I}}\,}}_{{{\,\mathrm{Sh}\,}}_U(X)[1/h]}(V) + R[P(U \oplus V)/M(V)][1/h]\right\} . \end{aligned}$$

There is a slight abuse of notation here: *M*(*V*) is a submodule of $P(U \oplus V)$, so $M(V)^*$ is naturally a quotient of $P(U \oplus V)^*$ rather than a submodule. But the projection $P(U \oplus V) \rightarrow P(U \oplus V)/M(V)$ admits a section (indeed, we have arranged things such that $P(U \oplus V)$ is isomorphic to the direct sum of the free *R*-modules *M*(*V*) and $P(U \oplus V)/M(V)$), and any section yields a section $M(V)^* \rightarrow P(U \oplus V)^*$. Two such sections differ by adding elements from $(P(U \oplus V)/M(V))^*$, which is contained in the second term above, so *N*(*V*) does not depend on the choice of section.

Recall from Sect. 5.2 that $V^* \mapsto M(V)^*$ is a polynomial functor $M^*$ of degree *d*.

#### Lemma 77

The association $V^* \mapsto N(V)$ is a polynomial subfunctor of $M^*$.

#### Proof

Let *A* be an *R*-algebra and take $V,W\in {{\,\mathrm{\mathbf {fgfMod}_R}\,}}$. Take $y'\in A\otimes N(V)$ and $\varphi ^*\in A\otimes {{\,\mathrm{Hom}\,}}(V^*,W^*)$ corresponding to $\varphi \in A \otimes {{\,\mathrm{Hom}\,}}(W,V)$. Then

$$\begin{aligned} A\otimes {{\,\mathrm{Hom}\,}}(M(W),M(V))&\cong A\otimes {{\,\mathrm{Hom}\,}}(M(V)^*,M(W)^*)\\&\cong {{\,\mathrm{Hom}\,}}_A(A \otimes M(V)^*,A\otimes M(W)^*). \end{aligned}$$

Denote the image of $M^*_{V^*,W^*,A}(\varphi ^*)=M_{W,V,A}(\varphi )$ in ${{\,\mathrm{Hom}\,}}_A(A \otimes M(V)^*,A\otimes M(W)^*)$ by $M_{W,V,A}(\varphi )^*$. We need to show that $M_{W,V,A}(\varphi )^*(y')\in A\otimes N(W)$. This condition is *A*-linear in $y'$, so we may assume that $y'=1 \otimes y$ with $y \in N(V)$.

Choose $A=R[x_1,\ldots ,x_n]$ and $\varphi =\sum _i x_i\otimes \varphi _i$ where the $\varphi _i$ form a basis of ${{\,\mathrm{Hom}\,}}(W,V)$. Then in particular we need that

$$\begin{aligned} M_{W,V,R[x_1,\ldots ,x_n]}\left( {\sum _i} x_i\otimes \varphi _i \right) ^*(1\otimes y)\in R[x_1,\ldots ,x_n]\otimes N(W). \end{aligned}$$

Conversely, by specializing the $x_i$ to $a_i\in A$ for any *R*-algebra *A*, this in fact suffices. As *M* is a subfunctor of *P*, we may here replace *M* by *P*.

Since *P*(*V*) is free, the *R*-linear map

$$\begin{aligned} P_{W,V,R[x_1,\ldots ,x_n]}\left( {\sum _i} x_i\otimes \varphi _i \right) ^*|_{P(V)^*}:P(V)^*\rightarrow R[x_1,\ldots ,x_n]\otimes P(W)^* \end{aligned}$$

induces a homomorphism $\Phi :R[P(V)]\rightarrow R[x_1,\ldots ,x_n]\otimes R[P(W)]$ of *R*-algebras. As taking the $p^e$-th power is additive, an element *z* is contained in $R[x_1,\ldots ,x_n]\otimes N(W)$ if and only if $z^{p^e}$ is contained in

$$\begin{aligned} R[x_1,\ldots ,x_n]\otimes ({{\,\mathrm{\mathcal {I}}\,}}_{{{\,\mathrm{Sh}\,}}_U(X)[1/h]}(W) + R[P(U \oplus W)/M(W)][1/h]). \end{aligned}$$

So we now need to show that $\Phi (y)^{p^e}=\Phi (y^{p^e})$ is contained in this latter set. Since $y\in N(V)$, we have $y^{p^e}=g_1+g_2$ for some $g_1\in {{\,\mathrm{\mathcal {I}}\,}}_{{{\,\mathrm{Sh}\,}}_U(X)[1/h]}(V)$ and $g_2\in R[P(U \oplus V)/M(V)][1/h]$. Now we note that $\Phi (g_1)\in R[x_1,\ldots ,x_n]\otimes {{\,\mathrm{\mathcal {I}}\,}}_{{{\,\mathrm{Sh}\,}}_U(X)[1/h]}(W)$ as in the proof of Lemma 66 and $\Phi (g_2)\in R[x_1,\ldots ,x_n]\otimes R[P(U \oplus W)/M(W)][1/h]$. So indeed

$$\begin{aligned} M_{W,V,R[x_1,\ldots ,x_n]}\left( {\sum _i} x_i\otimes \varphi _i \right) ^*(1\otimes y)\in R[x_1,\ldots ,x_n]\otimes N(W) \end{aligned}$$

holds. $$\square $$

#### Lemma 78

For every $V\in {{\,\mathrm{\mathbf {fgfMod}_R}\,}}$, every element of $M(V)^*$ has a nonzero *R*-multiple in *N*(*V*).

#### Proof

By [8, Lemma 25], any element *x* of $M(V)^*$ has $1 \otimes x^{p^e} \in K \otimes N(V) \subseteq K \otimes M(V)^*$; in the symbol $\subseteq$ we use that *M*(*V*), and hence $M(V)^*$, are free. Clearing denominators, we find that $r x^{p^e} \in M(V)^*$ for some nonzero $r \in R$. $\square$

#### Lemma 79

There exists a nonzero $r \in R$ such that $R[1/r] \otimes N(V) = R[1/r] \otimes M(V)^*$ holds for all $V \in {{\,\mathrm{\mathbf {fgfMod}_R}\,}}$.

#### Proof

Recall that the degree of the polynomial functor *M* is *d* and consider $V = R^d$. By Lemma 78 and the fact that *M*(*V*) is finitely generated, there exists a nonzero $r\in R$ such that $R[1/r]\otimes N(V) = R[1/r] \otimes M(V)^*$. The Friedlander–Suslin lemma, for polynomial functors over *R*[1/*r*], gives that then $R[1/r] \otimes N(V) = R[1/r] \otimes M(V)^*$ for every *V*. $\square$

We now replace *R* by the localisation *R*[1/*r*] and may henceforth assume that $N(V)=M(V)^*$.

### Proof of condition (2)

To establish condition (2) for (*P*, *R*, *X*), we will first prove an analogous statement for the localised shift.

#### Lemma 80

There exists a nonzero $r \in R$ such that the following holds for all $V\in {{\,\mathrm{\mathbf {fgfMod}_R}\,}}$: if $g\in R[P(U\oplus V)]$ vanishes identically on ${{\,\mathrm{Sh}\,}}_U(X)[1/h](V)(\overline{K})$, then *g* vanishes identically on ${{\,\mathrm{Sh}\,}}_U(X)[1/h](V)(\overline{K_\mathfrak {p}})$ for all primes $\mathfrak {p}\in {{\,\mathrm{Spec}\,}}(R[1/r])$.

#### Proof

Assume that $g\in R[P(U\oplus V)]$ vanishes identically on ${{\,\mathrm{Sh}\,}}_U(X)[1/h](V)(\overline{K})$. View *g* as a polynomial in the coordinates $x_i$ of $M(V)^*$ corresponding to a basis of *M*(*V*) with coefficients in $R[P(U \oplus V)/M(V)]$. By the conclusion of Sect. 6.9, we have $N(V) = M(V)^*$, which means that each $x_i^{p_e}$ is a sum of an element in $R[P(U \oplus V)/M(V)][1/h]$ and an element in the ideal of ${{\,\mathrm{Sh}\,}}_U(X)[1/h](V)$. We then find that also $g^{p^e}=g_1 + g_2$ with $g_1 \in R[P(U \oplus V)/M(V)][1/h]$ and $g_2 \in {{\,\mathrm{\mathcal {I}}\,}}_{{{\,\mathrm{Sh}\,}}_U(X)[1/h](V)}$. Let *Z* be the closure of the projection of ${{\,\mathrm{Sh}\,}}_U(X)[1/h]$ to $({{\,\mathrm{Sh}\,}}_U(P)/M)[1/h]$. Since both *g* and $g_2$ vanish identically on ${{\,\mathrm{Sh}\,}}_U(X)[1/h](V)(\overline{K})$, $g_1$ vanishes identically on $Z(V)(\overline{K})$. By the outer induction hypothesis, after a localisation that doesn’t depend on $g_1$ or on *V*, one concludes that $g_1$ vanishes identically on $Z(V)(\overline{K_\mathfrak {p}})$ for all $\mathfrak {p}\in {{\,\mathrm{Spec}\,}}(R)$. But then $g^{p^e}$, and hence *g* itself, vanish identically on ${{\,\mathrm{Sh}\,}}_U(X)[1/h](V)(\overline{K_{\mathfrak {p}}})$. $\square$

Now we can establish condition (2) of $\Sigma (R,P,X)$:

#### Proposition 81

There exists a nonzero $r \in R$ such that the following holds for all $V\in {{\,\mathrm{\mathbf {fgfMod}_R}\,}}$: if $g\in R[P(V)]$ vanishes identically on $X(V)(\overline{K})$, then *g* vanishes identically on $X(V)(\overline{K_\mathfrak {p}})$ for all primes $\mathfrak {p}\in {{\,\mathrm{Spec}\,}}(R[1/r])$.

#### Remark 82

For each fixed *V*, such an *r* exists by Proposition 44. Taking the product of such *r*’s, the same applies to a finite number of *V*’s, so we may restrict our attention to all *V* of sufficiently large rank; we will do this in the proof.

#### Proof of Proposition 81

By the inner induction hypothesis, after replacing *R* by a localisation *R*[1/*r*], we know that if $g \in R[P(V)]$ vanishes identically on $Y(V)(\overline{K})$, then it vanishes identically on $Y(V)(\overline{K_\mathfrak {p}})$ for all $\mathfrak {p}\in {{\,\mathrm{Spec}\,}}(R)$.

For any $V \in {{\,\mathrm{\mathbf {fgfMod}_R}\,}}$ and $\mathfrak {p}\in {{\,\mathrm{Spec}\,}}(R)$, define $Z(V)(\overline{K_{\mathfrak {p}}}):=X(V)(\overline{K_{\mathfrak {p}}}) \setminus Y(V)(\overline{K_{\mathfrak {p}}})$. It suffices to show that with a further localisation we achieve that for any $V \in {{\,\mathrm{\mathbf {fgfMod}_R}\,}}$, if $g \in R[P(V)]$ vanishes identically on all points of $Z(V)(\overline{K})$, then it vanishes identically on all points of $Z(V)(\overline{K_\mathfrak {p}})$ for all $\mathfrak {p}\in {{\,\mathrm{Spec}\,}}(R)$. In proving this, by Remark 82 above, we may assume that *V* has rank at least that of *U*. Hence we may replace *V* by $U \oplus V$.

Such a *g* that vanishes identically on $Z(U \oplus V)(\overline{K})$ vanishes, in particular, identically on ${{\,\mathrm{Sh}\,}}_U(X)[1/h](V)(\overline{K})$. Lemma 80 says that (after replacing *R* by a localisation that does not depend on *g* or *V*), *g* also vanishes identically on ${{\,\mathrm{Sh}\,}}_U(X)[1/h](V)(\overline{K_\mathfrak {p}})$ for all $\mathfrak {p}\in {{\,\mathrm{Spec}\,}}R$. This basic open is actually dense in $Z(U \oplus V)(\overline{K_\mathfrak {p}})$, as one sees as follows: $Z(U \oplus V)(\overline{K_\mathfrak {p}})$ is the image of the action

$$\begin{aligned} {{\,\mathrm{GL}\,}}(\overline{K_{\mathfrak {p}}} \otimes (U\oplus V)) \times {{\,\mathrm{Sh}\,}}_U(X)[1/h](V)(\overline{K_\mathfrak {p}}) \rightarrow X(U \oplus V)(\overline{K_\mathfrak {p}}). \end{aligned}$$

If the basic open were contained in the union of a proper subset of the irreducible components of $Z(U\oplus V)(\overline{K_\mathfrak {p}})$, then, by irreducibility of ${{\,\mathrm{GL}\,}}(\overline{K_{\mathfrak {p}}} \otimes (U\oplus V))$, so would the image of that action, a contradiction. Hence *g* then vanishes identically on $Z(V)(\overline{K_\mathfrak {p}})$ for all $\mathfrak {p}\in {{\,\mathrm{Spec}\,}}(R)$. $\square$

#### Remark 83

Note that, unlike *Y*, the *Z* defined in the proof is not a subset of *X* in the sense of Definition 61.

### Proof of the Noetherianity of *X*

Finally, we prove condition (1) of $\Sigma (R,P,X)$. Let $X=X_1 \supseteq X_2 \supseteq \cdots$ be a sequence of closed subsets of *X*. Recall from Sect. 6.8 that the intersections of the $X_i$ with *Y* stabilise. Now, consider again the projection ${{\,\mathrm{Sh}\,}}_U(P)[1/h] \rightarrow ({{\,\mathrm{Sh}\,}}_U(P)/M)[1/h]$. We let $Z_i'$ be the closure of the image of ${{\,\mathrm{Sh}\,}}_U(X_i)[1/h]$ in $({{\,\mathrm{Sh}\,}}_U(P)/M)[1/h]$. Since the polynomial functor $({{\,\mathrm{Sh}\,}}_U(P)/M)$ is smaller then *P*, we have Noetherianity for $({{\,\mathrm{Sh}\,}}_U(P)/M)[1/h]$ and therefore the sequence $Z_1' \supseteq Z_2' \supseteq \cdots$ stabilises. We now conclude from this that the sequence of ${{\,\mathrm{Sh}\,}}_U(X_i)[1/h]$’s also stabilises.

#### Lemma 84

Let $X''\subseteq X'\subseteq X$ be closed subsets, assume ${{\,\mathrm{Sh}\,}}_U(X'')[1/h]\subsetneq {{\,\mathrm{Sh}\,}}_U(X')[1/h]$ and let $Z''\subseteq Z'$ be the closures of their images in $({{\,\mathrm{Sh}\,}}_U(P)/M)[1/h]$. Then $Z''\subsetneq Z'$.

#### Proof

Since ${{\,\mathrm{Sh}\,}}_U(X'')[1/h]\subsetneq {{\,\mathrm{Sh}\,}}_U(X')[1/h]$, we have

$$\begin{aligned} {{\,\mathrm{Sh}\,}}_U(X'')[1/h](V)\subsetneq {{\,\mathrm{Sh}\,}}_U(X')[1/h](V) \end{aligned}$$

for some $V\in {{\,\mathrm{\mathbf {fgfMod}}\,}}_R$. This means that ${{\,\mathrm{\mathcal {I}}\,}}_{{{\,\mathrm{Sh}\,}}_U(X'')[1/h]}(V)\supsetneq {{\,\mathrm{\mathcal {I}}\,}}_{{{\,\mathrm{Sh}\,}}_U(X')[1/h]}(V)$. Let $g\in R[P(U\oplus V)][1/h]$ be an element of the former ideal that is not contained in the latter. Then the same holds for $g^{p^e}$. By the conclusion of Sect. 6.9, $g^{p^e}$ is a sum of an element $g_1$ in $R[P(U \oplus V)/M(V)][1/h]$ and an element $g_2$ of ${{\,\mathrm{\mathcal {I}}\,}}_{{{\,\mathrm{Sh}\,}}_U(X)[1/h]}(V)\subseteq {{\,\mathrm{\mathcal {I}}\,}}_{{{\,\mathrm{Sh}\,}}_U(X')[1/h]}(V)$. This means that $g_1$ is also an element of ${{\,\mathrm{\mathcal {I}}\,}}_{{{\,\mathrm{Sh}\,}}_U(X'')[1/h]}(V)$ not contained in ${{\,\mathrm{\mathcal {I}}\,}}_{{{\,\mathrm{Sh}\,}}_U(X')[1/h]}(V)$. Hence

$$\begin{aligned}&{{\,\mathrm{\mathcal {I}}\,}}_{{{\,\mathrm{Sh}\,}}_U(X'')[1/h]}(V)\cap R[P(U \oplus V)/M(V)][1/h]\supsetneq {{\,\mathrm{\mathcal {I}}\,}}_{{{\,\mathrm{Sh}\,}}_U(X')[1/h]}(V)\\&\quad \cap R[P(U \oplus V)/M(V)][1/h] \end{aligned}$$

holds. The former ideal of $R[P(U \oplus V)/M(V)][1/h]$ equals ${{\,\mathrm{\mathcal {I}}\,}}_{Z''}(V)$ and the latter equals ${{\,\mathrm{\mathcal {I}}\,}}_{Z'}(V)$. So $Z''(V)\subsetneq Z'(V)$ and hence $Z''\subsetneq Z'$. $\square$

By the lemma, the fact that the sequence of $Z_i'$ stabilises implies that the sequence of ${{\,\mathrm{Sh}\,}}_U(X_i)[1/h]$’s also stabilises. Now again, we write

$$\begin{aligned} Z_i(V)(\overline{K_{\mathfrak {p}}})=X_i(V)(\overline{K_{\mathfrak {p}}})\setminus Y(V)(\overline{K_{\mathfrak {p}}}) \end{aligned}$$

for all $V\in {{\,\mathrm{\mathbf {fgfMod}_R}\,}}$ and $\mathfrak {p}\in {{\,\mathrm{Spec}\,}}(R)$. We consider the descending sequence of $Z_i$’s. What is left to prove for the Noetherianity of *X* is the following result.

#### Lemma 85

The sequence $Z_1\supseteq Z_2\supseteq \cdots$ stabilises.

#### Proof

Let *m* be the rank of *U*. As in equation $(*)$ in [8, §2.9], we have

$$\begin{aligned} Z_i(U\oplus V)(\overline{K_{\mathfrak {p}}})= & {} \{p \in X_i(U \oplus V)(\overline{K_{\mathfrak {p}}}) \mid h (g(p)) \\&\quad \ne 0 \text { for some } g \in {{\,\mathrm{GL}\,}}(\overline{K_{\mathfrak {p}}}\otimes (U \oplus V))\}\\= & {} \bigcup _{g \in {{\,\mathrm{GL}\,}}(\overline{K_{\mathfrak {p}}}\otimes (U \oplus V))} g {{\,\mathrm{Sh}\,}}_U(X_i)[1/h](V)(\overline{K_{\mathfrak {p}}}) \end{aligned}$$

for every $\mathfrak {p}\in {{\,\mathrm{Spec}\,}}(R)$. So the sequence of $Z_i$’s restricted to $V\in {{\,\mathrm{\mathbf {fgfMod}_R}\,}}$ of rank $\ge m$ stabilizes. As the sequence of $X_i(R^k)$’s stabilizes for each $k\in \{0,\ldots ,m-1\}$ by Proposition 1, the unrestricted sequence of $Z_i$’s also stabilizes. $\square$

Since both the sequence of $X_i\cap Y$’s and $Z_i$’s stabilize, using Corollary 36, the sequence of $X_i$’s also stabilizes. So the closed subset *X* is Noetherian. This concludes the proof of condition (1) for (*R*, *P*, *X*) and hence the proof of Theorem 2.

### Dimension functions of closed subsets of polynomial functors

To illustrate that the proof method for Theorem 2 can be used to obtain further results on closed subsets of polynomial functors, we establish a natural common variant of Propositions 43 and 54. For each $\mathfrak {p}\in {{\,\mathrm{Spec}\,}}(R)$ define the function $f_\mathfrak {p}:{{\,\mathrm{{\mathbb Z}}\,}}_{\ge 0} \rightarrow {{\,\mathrm{{\mathbb Z}}\,}}_{\ge 0}$ as $f_\mathfrak {p}(n):=\dim (X(R^n)(\overline{K_\mathfrak {p}}))$.

#### Proposition 86

For each $\mathfrak {p}\in {{\,\mathrm{Spec}\,}}(R)$, $f_{\mathfrak {p}}(n)$ is a polynomial in *n* with integral coefficients for all $n \gg 0$. Furthermore, the map that sends $\mathfrak {p}$ to this polynomial is constructible.

#### Proof

(Proof sketch) Both statements follow by inductions identical to the one for Theorem 2, using that, in the most interesting induction step, for $n \ge m:={{\,\mathrm{rk}\,}}(U)$ the dimension of $X_{\overline{K_\mathfrak {p}}}(\overline{K_\mathfrak {p}}^n)$ is the maximum of the dimensions of $Y_{\overline{K_\mathfrak {p}}}(\overline{K_\mathfrak {p}}^n)$ and

$$\begin{aligned}({{\,\mathrm{Sh}\,}}_U(X)[1/h])_{\overline{K}_\mathfrak {p}}(\overline{K_{\mathfrak {p}}}^{n-m}).\end{aligned}$$

Furthermore, for the case where $X_{\overline{K_\mathfrak {p}}}$ is the pre-image of $X_{\overline{K_\mathfrak {p}}}'$, we use Proposition 54, and for the base case in the induction proof for the constructibility statement we use Proposition 43. $\square$

#### Example 87

Take $R={{\,\mathrm{{\mathbb Z}}\,}}$, take $P=S^3$, and let *X* be the closed subset defined as the image closure of the polynomial transformation $(S^1)^2 \rightarrow S^3, (v,w) \mapsto v^3 + w^3$; see Sect. 1.3 for similar polynomial transformations. Then $X_{\overline{K_\mathfrak {p}}}(\overline{K_\mathfrak {p}}^n)$ has dimension 2*n* for $\mathfrak {p}\ne (3)$ and dimension *n* for $\mathfrak {p}=(3)$, since in the latter case the set of cubes of linear forms is a linear subspace of the space of cubics. This is an instance of Proposition 86.

## References

[1] Akin K, Buchsbaum DA, Weyman J (1982). Schur functors and Schur complexes. Adv. Math..

[2] Ananyan T, Hochster M (2020). Small subalgebras of polynomial rings and Stillman’s conjecture. J. Am. Math. Soc..

[3] Blekherman, G., Hauenstein, J., Ottem, J.C., Ranestad, K., Sturmfels, B.: Algebraic boundaries of Hilbert’s SOS cones. Compos. Math. **148**(6), 1717–1735 (2012)

[4] Bik, A., Draisma, J., Eggermont, R.H.: Polynomials and tensors of bounded strength. Commun. Contemp. Math. **21 **(7), 1850062 (2019)

[5] Bik, A., Draisma, J., Eggermont, R.H., Snowden, A.: The geometry of polynomial representations **(preprint)**. arXiv:2105.12621

[6] Carter RW, Lusztig G (1974). On the modular representations of the general linear and symmetric groups. Math. Z..

[7] Derksen H, Eggermont RH, Snowden A (2017). Topological noetherianity for cubic polynomials. Algebra Number Theory.

[8] Draisma J (2019). Topological Noetherianity of polynomial functors. J. Am. Math. Soc..

[9] Draisma J, Lasoń M, Leykin A (2019). Stillman’s conjecture via generic initial ideals. Commun. Algebra.

[10] Eisenbud D (1995). Commutative Algebra with a View Toward Algebraic Geometry, Graduate Texts in Mathematics.

[11] Erman D, Sam SV, Snowden A (2019). Big polynomial rings and Stillman’s conjecture. Invent. Math..

[12] Erman D, Sam SV, Snowden A (2020). An equivariant Hilbert basis theorem. Math. Res. Lett..

[13] Erman D, Sam SV, Snowden A (2021). Generalizations of Stillman’s conjecture via twisted commutative algebra. Int. Math. Res. Not..

[14] Erman D, Sam SV, Snowden A (2021). Small projective spaces and Stillman uniformity for sheaves. Algebr. Geom..

[15] Friedlander E, Suslin A (1997). Cohomology of finite group schemes over a field. Invent. Math..

[16] Green, J.A.: Polynomial representations of $$\text{GL}_n$$. With an appendix on Schensted correspondence and Littelmann paths by K. Erdmann, J. A. Green and M. Schocker, Lecture Notes in Mathematics, vol. 830. Springer, New York (2007)

[17] Kazhdan D, Ziegler T (2018). On ranks of polynomials. Algebras Represent. Theory.

[18] Kazhdan, D., Ziegler, T.: Extending weakly polynomial functions from high rank varieties **(preprint)**. arXiv:1808.09439

[19] Nagpal, R., Sam, S.V., Snowden, A.: Noetherianity of some degree two twisted commutative algebras. Selecta Math. (N.S.) **22**(2), 913–937 (2016)

[20] Peeva I, Stillman M (2009). Open problems on syzygies and Hilbert functions. J. Commut. Algebra.

[21] Pirashvili, T.: Polynomial functors over finite fields. Astérisque **276**, 369–388, Exp. no. 877 (2002)

[22] Roby, N.: Lois polynomes et lois formelles en théorie des modules. Annales scientifiques de l’É.N.S. 3e série, tome 80(3), 213–348 (1963)

[23] Sam SV, Snowden A (2016). $$\text{ GL }$$-equivariant modules over polynomial rings in infinitely many variables. Trans. Am. Math. Soc..

[24] Sam, S.V., Snowden, A.: $$\text{ GL }$$-equivariant modules over polynomial rings in infinitely many variables. II. Forum Math. Sigma **7**, Paper no. e5 (2019)

[25] Sam, S.V., Snowden, A.: $$\text{ Sp }$$-equivariant modules over polynomial rings in infinitely many variables. Trans. Am. Math. Soc. **(to appear)**

[26] Sawin, W., Tao, T.: Notes on the slice rank of tensors. https://terrytao.wordpress.com/2016/08/24/notes-on-the-slice-rank-of-tensors/

[27] Touzé, A.: Foncteurs strictement polynomiaux et applications, Habilitation Thesis (2014). http://math.univ-lille1.fr/~touze/NotesRecherche/Habilitation_A_Touze.pdf

